# Efficacy of Curcumin in Neurodegenerative Diseases: From Pharmacokinetic Barriers to Advanced Delivery Systems

**DOI:** 10.3390/ph19091405

**Published:** 2026-09-06

**Authors:** Alejandra Castello-Guillen, Marta Garrido-Reig, Jordi Caplliure-Llopis, María Jesús Vega-Bello, Celia Almela, José Enrique de la Rubia Ortí

**Affiliations:** 1 Department of Nutrition, Faculty of Medicine and Health Sciences, Catholic University of Valencia San Vicente Mártir, 46003 Valencia, Spain; alejandra.castello@mail.ucv.es (A.C.-G.); martagr181@gmail.com (M.G.-R.); mj.vega@ucv.es (M.J.V.-B.); celia.almela@ucv.es (C.A.); 2 Emergency Medical Services of the Valencian Community (SESCV), 46009 Valencia, Spain; 3 Institute for Research on Musculoskeletal Disorders, Catholic University of Valencia San Vicente Mártir, Quevedo, 2, 46003 Valencia, Spain; 4 Department of Basic Biomedical Sciences, Catholic University of Valencia San Vicente Mártir, 46001 Valencia, Spain

**Keywords:** curcumin, neurodegenerative disease, blood–brain barrier, bioavailability, drug delivery systems, nanoparticles, liposomes, exosomes

## Abstract

**Background and Objectives:** The main neurodegenerative diseases (NDs)—Alzheimer’s disease (AD), Parkinson’s disease (PD), multiple sclerosis (MS), and amyotrophic lateral sclerosis (ALS)—represent a growing global health burden with no available disease-modifying therapies. Curcumin, a polyphenol from *Curcuma longa*, is a promising candidate owing to its pleiotropic antioxidant, anti-inflammatory, and neuroprotective profile observed mainly in preclinical models, but the poor oral bioavailability (<1%) and negligible BBB penetration (<0.1%) have substantially limited curcumin’s clinical translation. The objective of this work was to critically examine the therapeutic potential of curcumin in NDs, focusing on advanced drug delivery systems (DDSs) designed to overcome its pharmacokinetic barriers. **Methods:** This is a narrative, non-systematic review of PubMed/MEDLINE, Scopus, and Web of Science. The review is organized around five complementary thematic areas selected to span the full translational pipeline of curcumin in neurodegeneration, from mechanistic rationale to clinical applicability: (1) molecular mechanisms, addressing the pleiotropic activities that justify therapeutic interest; (2) pharmacokinetic barriers, the principal obstacle to clinical translation; (3) the evolution of drug delivery systems (DDSs), documenting the technological strategies developed to overcome these barriers; (4) disease-specific applications, evaluating the available evidence across the four main NDs; and (5) translational limitations, identifying the methodological and regulatory gaps that must be closed to enable clinical implementation. **Results:** Curcumin exhibits neuroprotective activity in preclinical models of the four NDs analysed, acting on six interconnected mechanisms and the gut–brain axis. Four generations of DDSs have been developed, from phytosomes and clinically used lipid dispersions (Meriva^®^, BCM-95^®^, Longvida^®^, and Theracurmin^®^) to fourth-generation systems (biomimetic nanoparticles, MOFs, microneedles, 3D scaffolds, hydrogels, and carbon dots) that substantially increase the bioavailability in preclinical studies. Combination strategies, such as curcumin with resveratrol and dutasteride, show preliminary clinical signals in ALS. However, clinical translation remains limited: over 80% of positive animal findings have not been replicated in humans, formulation characterization is frequently incomplete, and most trials lack CNS-exposure biomarkers. Importantly, most of the reported bioavailability claims are based on total curcumin measurements (parent aglycone plus its inactive Phase II conjugates) rather than the active aglycone alone, a methodological limitation that should be considered when interpreting the magnitude of the bioavailability improvements reported for novel formulations. **Conclusions:** Curcumin exhibits pleiotropic neuroprotective activity in preclinical models of AD, PD, MS, and ALS, mediated by interconnected antioxidant, anti-inflammatory, anti-amyloidogenic, mitochondrial, and gut–brain axis mechanisms. However, its poor systemic bioavailability (<1%), minimal blood–brain barrier penetration, and extensive first-pass metabolism have limited clinical translation. Advanced drug delivery systems (including lipid-based carriers (liposomes, solid lipid nanoparticles, and nanostructured lipid carriers), polymeric nanoparticles (PLGA and chitosan), and bioinspired vesicles (exosomes)) are essential in order to overcome these barriers. Nevertheless, the formulation heterogeneity, limited long-term safety data, and reliance on preclinical models remain major obstacles; a definitive clinical translation will therefore require well-characterized formulations validated in phase II/III trials with cerebrospinal fluid exposure biomarkers, the pharmacokinetic monitoring of active aglycone (rather than total curcumin including inactive conjugates), and adaptive trial designs in neurological populations.

## 1. Introduction

Neurodegenerative diseases (NDs)—including Alzheimer’s disease (AD), Parkinson’s disease (PD), multiple sclerosis (MS), and amyotrophic lateral sclerosis (ALS)—cause the progressive loss of specific neuronal populations, resulting in cognitive and motor decline [1,2]. Despite decades of research, therapeutic options remain largely limited to symptomatic relief, and no disease-modifying treatment has yet demonstrated a consistent efficacy across the major NDs. Current therapies, including cholinesterase inhibitors, dopaminergic agents, immunomodulators, and antisense oligonucleotides, target isolated pathogenic mechanisms and provide a modest or transient clinical benefit. The World Health Organization (WHO) estimates that more than 55 million people worldwide are currently living with dementia, a figure projected to nearly triple by 2050 due to population ageing [3]. This widening therapeutic gap underscores the urgent need for pleiotropic interventions capable of addressing the convergent molecular mechanisms underlying neurodegeneration, which remain insufficiently targeted by current single-mechanism strategies [4,5]. It should be noted that most of the reported bioavailability values for curcumin (including those for novel formulations) quantify total curcumin (parent aglycone plus its inactive Phase II conjugates) rather than the active aglycone alone, which may substantially overestimate the true systemic exposure to the pharmacologically active compound.

From a pathophysiological perspective, NDs share several convergent molecular mechanisms that ultimately drive neuronal dysfunction. These include oxidative stress, to which neurons are particularly susceptible owing to their high metabolic demand and limited antioxidant defenses; mitochondrial dysfunction; chronic neuroinflammation driven by the sustained activation of microglia and astrocytes; the accumulation of misfolded or aggregated proteins, including β-amyloid, hyperphosphorylated tau, α-synuclein, and TAR DNA-binding protein 43 (TDP-43); and excitotoxicity [1,4]. This remarkable convergence of pathogenetic pathways has fueled growing interest in pleiotropic natural compounds capable of simultaneously modulating multiple therapeutic targets, thereby offering a promising alternative to the traditional single-target pharmacological approach [5,6].

**Figure 1:**
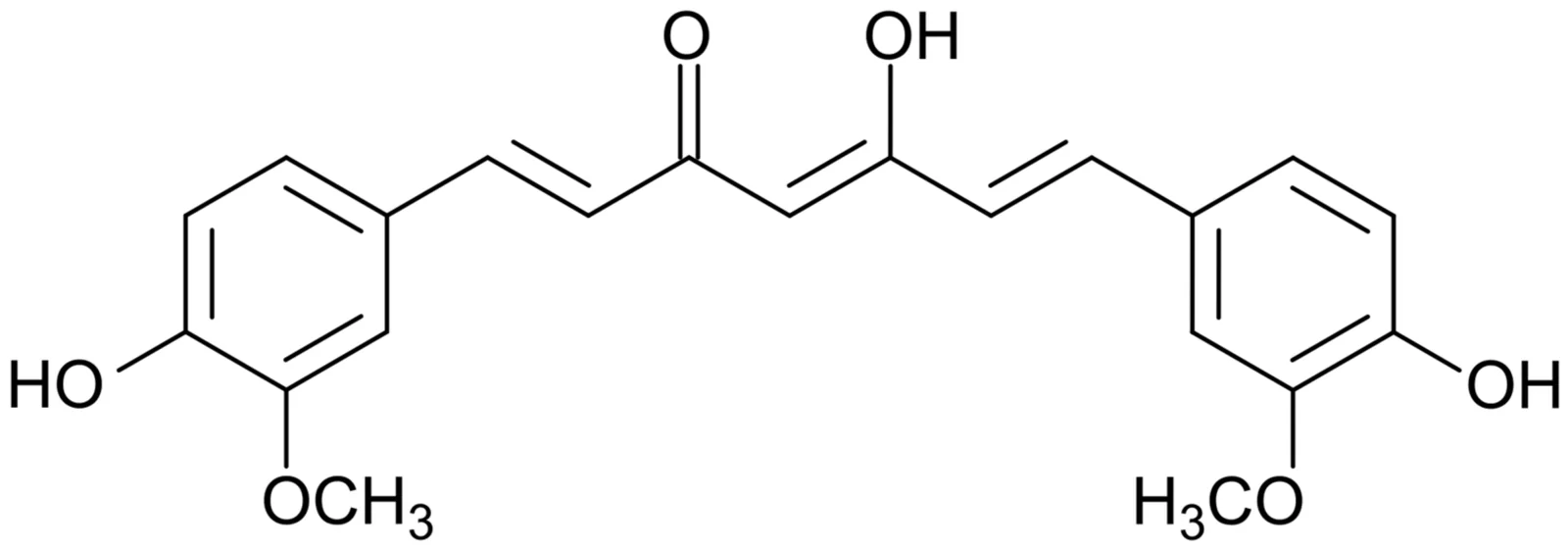
Chemical structure of curcumin. The molecule consists of two 4-hydroxy-3-methoxyphenyl (vanillyl) aromatic rings connected by a seven-carbon linker (heptadiene-3,5-dione) bearing a β-diketone moiety that undergoes keto–enol tautomerism in solution. This conjugated backbone, together with the two phenolic hydroxyl groups, underlies both the antioxidant activity and the poor pharmacokinetic profile of native curcumin (low aqueous solubility, logP ≈ 3.0, and extensive first-pass metabolism). MW 368.38 g/mol; molecular formula C_21_H_20_O_6_.

In this framework, curcumin, a hydrophobic polyphenol extracted from the rhizome of *Curcuma longa*, has attracted particular interest owing to its broad spectrum of biological activities (Figure 1)—including antioxidant, anti-inflammatory, anti-apoptotic, anti-amyloidogenic, and neuroprotective effects—and to a favourable short-term safety profile, with single oral doses of up to 12 g being well-tolerated in healthy volunteers [6,7,8]. However, it should be emphasized that this tolerability does not translate into pharmacologically meaningful systemic or central exposure, since native oral curcumin exhibits <1% bioavailability and minimal blood–brain barrier penetration (see Section 3) [9,10].

However, the clinical translation of curcumin is severely limited by a set of well-characterized pharmacokinetic barriers—low aqueous solubility, limited intestinal absorption, extensive first-pass metabolism (mainly glucuronidation and sulfation), poor systemic bioavailability, and, most critically, minimal blood–brain barrier (BBB) penetration [9,10]. These constraints largely explain the consistent failure of clinical trials with native curcumin to reproduce the encouraging preclinical findings, and have directly driven the development of advanced drug delivery systems (DDSs) specifically designed to overcome them [6,9,10,11].

This narrative review provides a comprehensive and critical overview of the therapeutic potential of curcumin in neurodegenerative diseases, with particular emphasis on DDSs currently under investigation. The review is organized as follows: Section 2 summarizes the molecular mechanisms of neuroprotection; Section 3 describes the pharmacokinetic barriers of native curcumin; Section 4 analyses the four generations of drug delivery systems; Section 5 reviews disease-specific applications in AD, PD, MS, and ALS; and Section 6 discusses the translational limitations and future directions required to enable clinical implementation. Overall, the evidence reviewed here indicates that advanced DDSs are essential to unlock curcumin’s therapeutic potential in neurodegenerative disease. Definitive clinical translation will, however, require well-characterized formulations validated in phase II/III trials with cerebrospinal fluid exposure biomarkers, a standardized pharmacokinetic assessment, and adaptive trial designs in neurological populations.

This narrative review is based on a non-systematic literature search conducted in PubMed/MEDLINE, Scopus, and Web of Science between January 2010 and early 2026, using combinations of the terms “curcumin”, “neurodegenerative disease”, “Alzheimer’s disease”, “Parkinson’s disease”, “multiple sclerosis”, “amyotrophic lateral sclerosis”, “blood-brain barrier”, “bioavailability”, “drug delivery system”, “nanoparticles”, “liposomes”, and “exosomes”, together with Boolean operators (AND, OR) to refine the results. The selection criteria included original preclinical and clinical studies, systematic reviews, and meta-analyses published in English (or with an English abstract); non-English literature was not formally searched but is referenced where relevant translation is available, with priority given to recent clinical evidence and high-impact translational studies; reference lists of relevant articles were manually screened to identify additional pertinent publications. The literature screening was carried out by two people, who independently reviewed titles and abstracts; any disagreements were resolved by discussion with the corresponding authors. Given the narrative, non-systematic scope of this work, no formal quality appraisal or risk-of-bias assessment using validated tools (e.g., AMSTAR 2, ROBIS) was performed; instead, potential bias was mitigated by cross-checking key references across multiple databases and prioritizing peer-reviewed studies with a robust methodology.

## 2. Molecular Mechanisms Underlying the Neuroprotective Effects of Curcumin

**Figure 2:**
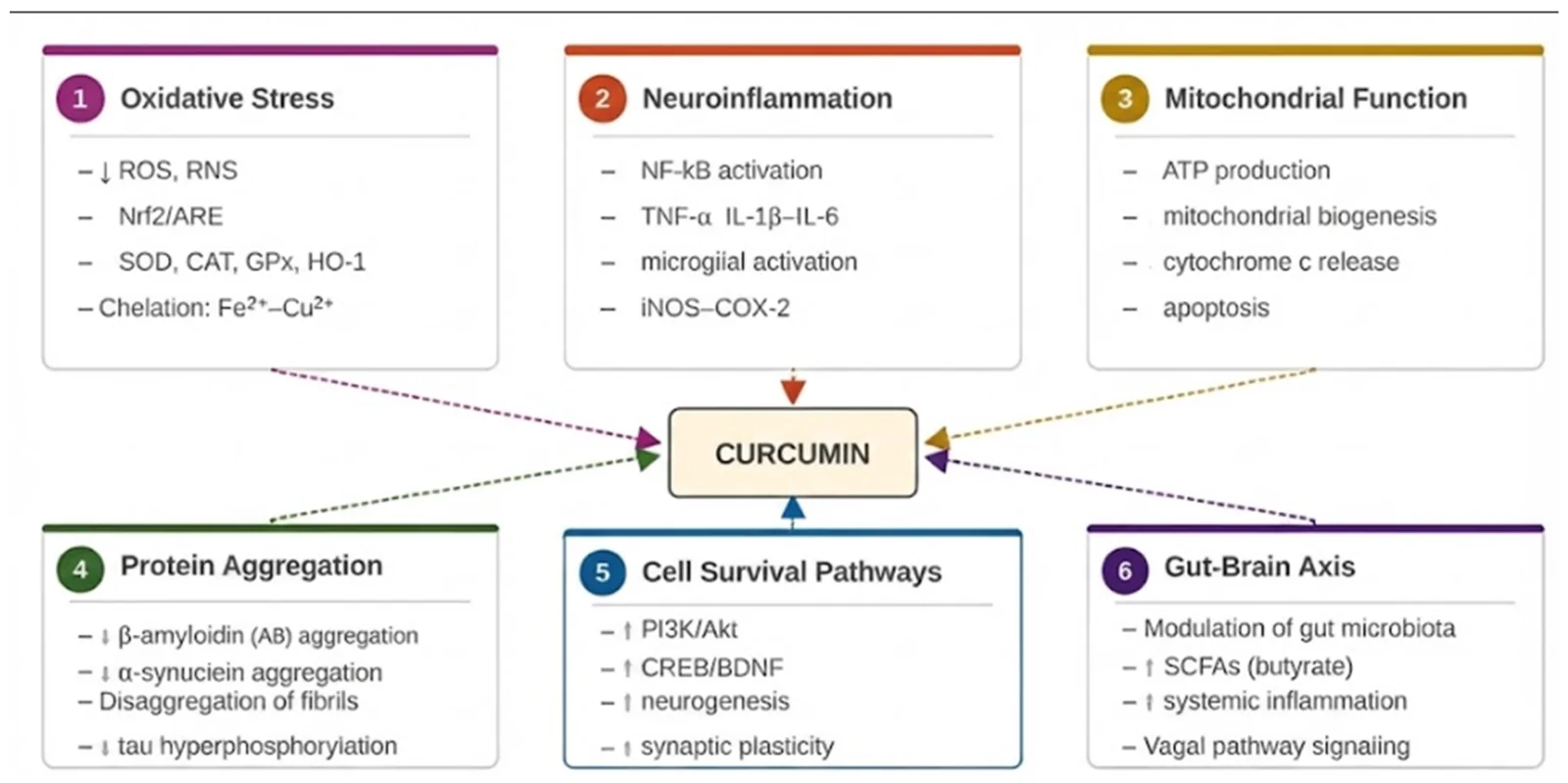
Multitarget mechanisms of curcumin in neurodegeneration. Curcumin exerts neuroprotective effects through six interconnected molecular pathways: it reduces oxidative stress by activating the Nrf2/ARE pathway and upregulating endogenous antioxidant defences (SOD, CAT, and GPx); inhibits neuroinflammation by suppressing NF-κB signalling and microglial activation, an effect that, in animal models, correlates with reduced neuronal loss in the hippocampus and cortex; improves mitochondrial function and cellular bioenergetics; interferes with the aggregation of pathological proteins (β-amyloid, α-synuclein, and tau), with reductions in aggregate load associated with attenuated synaptic deficits and improved cognitive performance in transgenic mouse models; activates cell survival pathways (PI3K/Akt and CREB/BDNF), promoting neurogenesis and synaptic plasticity; and modulates the gut–brain axis through effects on microbiota composition and short-chain fatty acid production. Abbreviations: ARE, antioxidant response element; BDNF, brain-derived neurotrophic factor; CAT, catalase; CREB, cAMP response element-binding protein; GPx, glutathione peroxidase; NF-κB, nuclear factor kappa B; Nrf2, nuclear factor erythroid 2-related factor 2; PI3K/Akt, phosphoinositide 3-kinase/protein kinase B; SOD, superoxide dismutase.

Following the introduction of the clinical relevance of neurodegenerative diseases and the therapeutic promise of curcumin, it is appropriate to briefly review the molecular mechanisms through which this polyphenol exerts its neuroprotective effects. This knowledge is essential for understanding why the pharmacokinetic limitations of curcumin (discussed in the following section) represent such a critical obstacle to its clinical translation, and why the development of DDSs (addressed in Section 4) has become the predominant strategy for unlocking its therapeutic potential. The principal mechanisms of action of curcumin for which consistent preclinical evidence is available are summarized below. These mechanistic axes are summarized in Figure 2.

### 2.1. Modulation of Signaling Pathways Involved in Neuronal Survival and Cell Death

Curcumin modulates several intracellular signalling pathways that are critical for neuronal survival and function. In the context of inflammation, curcumin inhibits the activation of nuclear factor kappa B (NF-κB), a master regulator of the inflammatory response, thereby reducing the production of pro-inflammatory cytokines (TNF-α, IL-1β, and IL-6) and microglial activation [12,13]; in animal models of neurodegeneration, this anti-inflammatory effect correlates with reduced neuronal loss in the hippocampus and cortex, supporting a downstream neuroprotective consequence. In terms of antioxidant defence, curcumin activates the Nrf2/ARE (nuclear factor erythroid 2-related factor 2/antioxidant response element) pathway, which upregulates the expression of three key antioxidant enzymes: (1) superoxide dismutase (SOD), which catalyses the dismutation of superoxide anions into hydrogen peroxide; (2) catalase (CAT), which decomposes hydrogen peroxide into water and oxygen; and (3) glutathione peroxidase (GPx), which reduces hydrogen peroxide and lipid hydroperoxides, protecting membrane lipids from oxidative damage. Together, these three enzymes form the first line of cellular defence against reactive oxygen species (ROS), and their coordinated induction translates in vivo into reduced levels of oxidative damage markers (e.g., malondialdehyde and 4-hydroxynonenal) and improved behavioural outcomes in models of AD and PD [12,13]. Regarding neuronal survival, curcumin activates the PI3K/Akt (phosphoinositide 3-kinase/protein kinase B) and CREB/BDNF (cAMP response element-binding protein/brain-derived neurotrophic factor) signalling pathways, promoting synaptic plasticity, neurogenesis, and cell survival, which, in rodent models, translates into an improved performance in spatial learning and memory tasks [13,14]. Furthermore, curcumin directly inhibits the aggregation of β-amyloid and α-synuclein and modulates tau phosphorylation, reducing the burden of pathological protein aggregates that characterize AD and PD; in transgenic mouse models, this reduction in aggregate load has been associated with attenuated synaptic deficits and improved cognitive performance [8,11,14].

### 2.2. Modulation of Programmed Cell Death Pathways

Apoptosis, Autophagy, and Ferroptosis:

Beyond the canonical neuroprotective signalling pathways described above, curcumin modulates several evolutionarily conserved cell death programs that are increasingly recognized as central to neurodegeneration: (1) Apoptosis: Curcumin shifts the BCL-2/BAX ratio in favour of anti-apoptotic signalling, attenuates mitochondrial outer-membrane permeabilization, and reduces caspase-3 and caspase-9 activation in neurons exposed to amyloid-β, 1-methyl-4-phenylpyridinium (MPP+), or SOD1-G93A conditions, preserving the cellular integrity and synaptic function [13]. (2) Autophagy: Curcumin induces autophagy through the coordinated inhibition of the AKT/mTOR axis and activation of BECN1 (Beclin-1), promoting the lipidation of LC3 (LC3-I → LC3-II) and the clearance of damaged mitochondria (mitophagy) and protein aggregates via the p62/SQSTM1 cargo receptor, an effect that, in transgenic AD and PD mouse models, reduces plaque and Lewy-body burden and improves behavioural outcomes [15]. (3) Ferroptosis: Curcumin acts as a lipid-peroxidation inhibitor in neurons by upregulating the selenoenzyme GPX4 (glutathione peroxidase 4), chelating labile iron pools via its β-diketone moiety, and suppressing the Fenton-driven accumulation of phospholipid hydroperoxides; in models of traumatic brain injury, ischemic stroke, and MPTP-induced parkinsonism, these actions reduce ferroptotic neuronal loss and preserve motor and cognitive function, although it should be noted that, in cancer cells, the same iron-chelating and ROS-generating chemistry can paradoxically promote ferroptosis as a therapeutic strategy [16]. Together, these three pathways expand the conceptual framework for curcumin’s neuroprotective activity beyond simple antioxidant/anti-inflammatory effects, and they help explain the breadth of its reported activity across multiple disease models.

### 2.3. Peripheral Actions and Modulation of the Gut–Brain Axis

Beyond its direct effects on the central nervous system (CNS), curcumin exerts peripheral actions that may contribute significantly to its neuroprotective effects. Chronic low-grade systemic inflammation, a hallmark of ageing and many NDs, is a well-recognized driver of neurodegenerative progression. When administered orally, curcumin reduces systemic inflammatory markers, including C-reactive protein (CRP), tumour necrosis factor-alpha (TNF-α), and interleukin-6 (IL-6), in numerous clinical studies, suggesting a systemic anti-inflammatory effect that may indirectly benefit neuronal function [12,17]. Moreover, curcumin modulates the composition of the gut microbiota by increasing the abundance of short-chain fatty acid (SCFA)-producing bacteria—primarily butyrate- and propionate-producing species—while reducing the abundance of pro-inflammatory bacteria. This shift has been associated with a reduced intestinal permeability (‘leaky gut’), lower circulating endotoxin (LPS) levels, and decreased systemic inflammation, which, together, may indirectly attenuate microglial activation in the brain [18,19]. Importantly, although plasma levels of the parent aglycone curcumin remain negligible, gut bacteria biotransform curcumin into bioactive metabolites such as tetrahydrocurcumin, dihydroferulic acid, and ferulic acid, which retain antioxidant and anti-inflammatory properties distinct from those of the parent compound [20]. Notably, tetrahydrocurcumin (the principal bacterial reduction product of curcumin) has been shown to possess equal or superior free-radical-scavenging activity to the parent compound and to attenuate Aβ oligomer-induced neurotoxicity, microglial activation, and neuronal apoptosis via Nrf2 pathway activation in rodent models, providing a plausible mechanism for central neuroprotection despite the negligible systemic aglycone exposure. As a representative example, in the MPTP mouse model of Parkinson’s disease, oral curcumin reshaped the gut microbiota and elevated SCFA (butyrate and propionate) levels, an effect that correlated with an attenuated microglial activation and the preservation of dopaminergic neurons in the substantia nigra, providing a plausible mechanistic basis for the central effects despite the minimal systemic aglycone exposure [18]. These microbiota- and metabolite-mediated changes, in turn, influence the intestinal barrier integrity, systemic inflammation, and brain function through the gut–brain axis, and are particularly relevant for interpreting the biological effects of oral curcumin formulations, which, despite their limited systemic bioavailability, may exert pharmacological activity through these indirect peripheral mechanisms [10,12,18].

## 3. Pharmacokinetic Limitations of Native Curcumin

**Figure 3:**
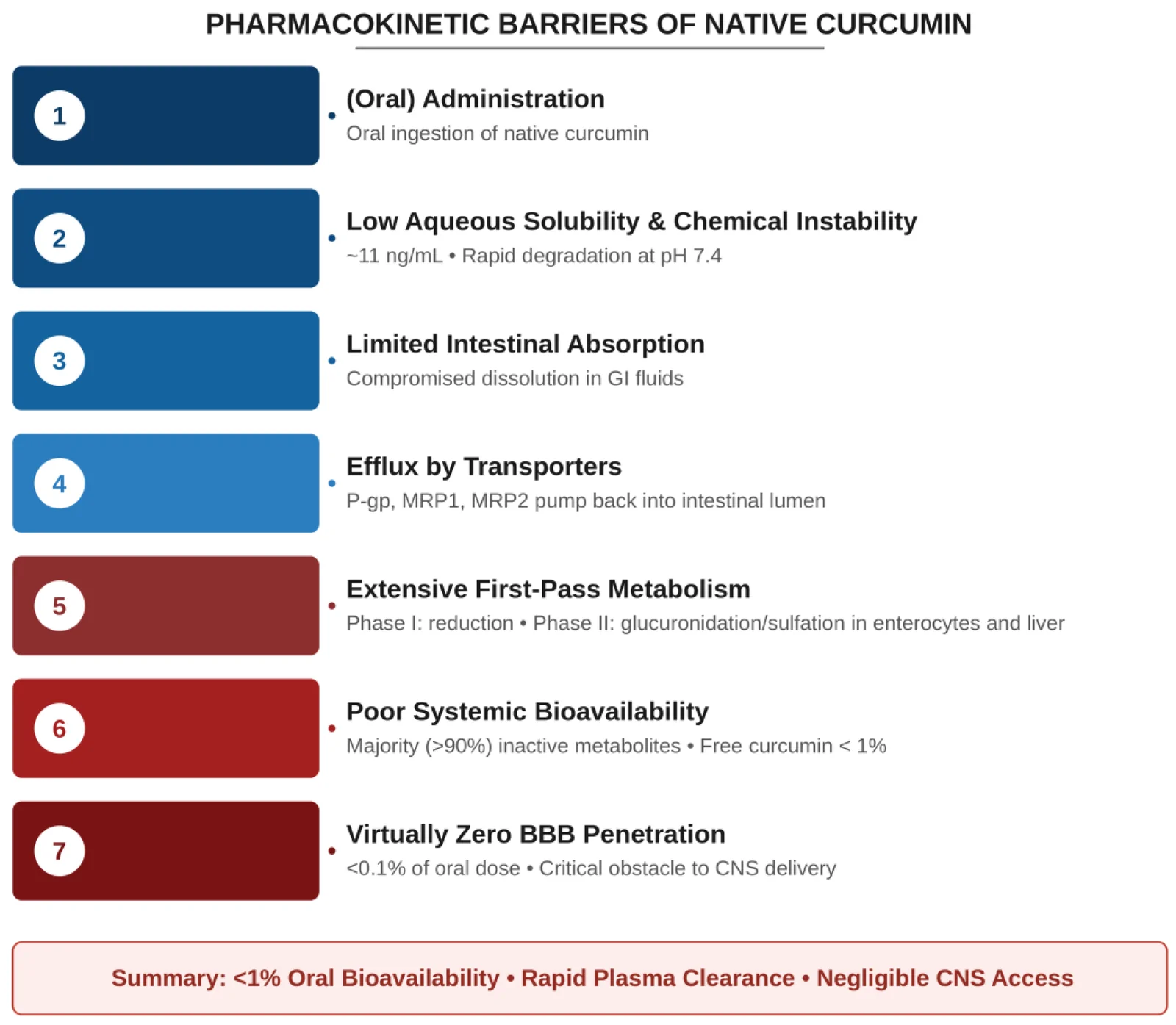
Pharmacokinetic barriers of native curcumin. Orally administered curcumin faces a sequence of progressive barriers that collectively limit its systemic and central exposure: its aqueous solubility is only ~11 ng/mL (~30 nM), at least 1000-fold lower than that of typical oral drugs; intestinal absorption is poor and limited by P-gp and MRP efflux; extensive first-pass hepatic metabolism (glucuronidation and sulfation) converts more than 90% of the dose into inactive conjugates; the resulting absolute oral bioavailability is below 1%; and blood–brain barrier penetration is virtually zero, with less than 0.1% of the oral dose reaching brain tissue. The cumulative effect is negligible systemic and central exposure of the active aglycone.

The pharmacokinetic profile of orally administered curcumin is determined by a series of intrinsic physicochemical properties that collectively limit its systemic exposure and, potentially, its access to the CNS. These limitations have been extensively described in preclinical and clinical pharmacokinetic studies and represent a major challenge that DDSs must overcome to enhance the therapeutic potential of curcumin in neurological disorders [9,10]. For context, the aqueous solubility of curcumin (~11 ng/mL) is at least 1000-fold lower than that of most orally bioavailable drugs (typically 10–1000 µg/mL), placing it firmly in the ‘poorly soluble’ category of the Biopharmaceutics Classification System (BCS). These barriers are summarized in Figure 3.

### 3.1. Poor Aqueous Solubility and Physicochemical Properties

Curcumin is a highly hydrophobic molecule with an octanol/water partition coefficient (log P) of approximately 3.2, resulting in an estimated aqueous solubility of only 11 ng/mL (~30 nM), under physiological conditions. This pronounced hydrophobicity limits its dissolution in gastrointestinal fluids and, consequently, its passive absorption across the intestinal epithelium. In addition, curcumin is highly susceptible to degradation under alkaline conditions, exhibiting a half-life of approximately 10 min in phosphate buffer at pH 7.4 [21]. These physicochemical properties represent the first bottleneck that any formulation must address, since, without adequate solubilization and protection against degradation, subsequent processes (absorption and distribution) are inevitably compromised [10,22]. The DDS-based strategies developed to address each of these physicochemical limitations—low aqueous solubility and chemical instability under physiological pH—are reviewed in detail in Section 4 (lipid-based, polymeric, inorganic, and stimuli-responsive systems) and quantitatively compared in the new Table 3 (DDS solubilization strategies and reported bioavailability enhancements).

### 3.2. Limited Intestinal Absorption and Efflux Transporters

Even when solubilized, curcumin exhibits poor intestinal absorption, partly due to its recognition by intestinal efflux transporters such as P-glycoprotein (P-gp) and multidrug resistance-associated proteins (MRP1 and MRP2) [8]. Animal studies have shown that the oral administration of curcumin at a dose of 2 g/kg in rats improves the systemic bioavailability of curcumin by as much as 154% and produces a maximum serum concentration (Cmax) of only 1.35 ± 0.23 μg/mL [23]. In a separate pharmacokinetic study, the intravenous administration of 10 mg/kg produced a Cmax of 0.36 ± 0.05 μg/mL and an estimated absolute oral bioavailability of approximately 1%, highlighting the poor systemic availability of native curcumin after oral administration [24]. In humans who ingested 2 g of pure curcumin powder after fasting, plasma curcumin was less than 10 ng/mL 1 h post-dose [25]. In the same study, the co-ingestion of curcumin with 20 mg of piperine appeared to increase the curcumin bioavailability by 2000%. In a study of oral curcumin, patients with preinvasive malignant or high-risk premalignant conditions of the bladder, skin, cervix, stomach, or oral mucosa received 0.5–8 g curcumin by mouth daily for 3 months [22]. Plasma curcumin concentrations were found to peak 1–2 h after intake and gradually declined within 12 h. The 8 g/day dose resulted in a peak serum concentration of 1.75 ± 0.80 M. When micronized curcumin was administered orally with orange juice at doses of 50–200 mg to 18 healthy volunteers, it was not detected in plasma at or above the limit of quantitation (approximately 0.63 ng/mL). To summarize, it appears that a low systemic bioavailability following oral dosing is consistent with the findings in preclinical models; efficient first-pass and some degree of intestinal metabolism of curcumin, particularly glucuronidation and sulfation, might explain its poor systemic availability when administered via the oral route. These findings explain why most of the ingested curcumin (>90%) does not reach the systemic circulation in its active form but is, instead, excreted in the faeces or converted into inactive conjugated metabolites [10,22,26].

### 3.3. Extensive First-Pass Metabolism

Curcumin undergoes particularly extensive first-pass metabolism involving intestinal, hepatic, and renal enzymes. During Phase I metabolism, NADPH-dependent reductases present in enterocytes and hepatocytes reduce the double bonds of curcumin, generating a series of reduced metabolites, including dihydrocurcumin, tetrahydrocurcumin, hexahydrocurcumin, and octahydrocurcumin [10,22]. These metabolites partially retain the biological activity of the parent compound. During Phase II metabolism, curcumin and its Phase I metabolites undergo extensive conjugation by sulfotransferases (SULT1A1 and SULT1A3 in the cytosol) and microsomal uridine 5′-diphospho-glucuronosyltransferases (UGTs), generating highly polar glucuronide and sulfate conjugates that are virtually devoid of biological activity and are rapidly eliminated via the bile and urine [10,26]. Intestinal-microbiota-mediated metabolism adds a further layer of complexity, as commensal bacteria can transform curcumin into metabolites such as dihydroferulic acid and ferulic acid, which exhibit biological activity and bioavailability profiles distinct from those of the parent compound [18]. Collectively, this extensive metabolism drastically reduces the amount of free curcumin available to exert pharmacological effects. Furthermore, a recent pharmacokinetic study has indicated that most claims regarding the bioavailability of commercial formulations are based on measurements of inactive conjugates, which may have led to an overestimation of the actual improvement in the exposure to the active compound [10,26]. The principal Phase I and Phase II metabolites of curcumin, together with their relative abundance and biological activity, are summarized in Table 1. Importantly, a key methodological caveat applies to almost all reported pharmacokinetic data: most bioavailability claims in the literature (including those for novel commercial formulations) measure total curcumin (i.e., the sum of free aglycone plus its inactive Phase II conjugates) rather than the active aglycone alone. Because curcumin glucuronide and sulfate conjugates are virtually devoid of pharmacological activity, this measurement artifact tends to substantially overestimate the systemic exposure to the active compound, and any interpretation of ‘improved bioavailability’ should therefore be made with caution [10,26].

### 3.4. Limited Penetration Across the Blood–Brain Barrier (BBB)

The BBB represents the most critical obstacle to curcumin delivery to the CNS. The BBB is a highly selective interface composed of tightly connected endothelial cells, pericytes, astrocytes, and a basement membrane, which strictly limits the passage of substances from the bloodstream into the brain parenchyma [1]. Owing to its hydrophobicity and relatively high molecular weight (~368 g/mol), curcumin exhibits a very limited capacity for passive diffusion across the BBB, and its concentration in brain tissue following systemic administration at standard doses is virtually undetectable, as demonstrated in preclinical studies using radiolabelled curcumin [9,10]. This limitation has been recognized as the principal bottleneck in the development of effective treatments for NDs based on natural compounds and has driven the development of active targeting strategies and DDSs specifically designed to cross or modulate the BBB [11,12]. Recent studies have demonstrated that even curcumin formulations with a high systemic bioavailability do not guarantee significant brain accumulation unless they incorporate explicit BBB-crossing strategies, highlighting the need to integrate both an improved bioavailability and brain-targeting capability into the design of DDSs [9,11].

## 4. Advanced Curcumin-Based Drug Delivery Systems (DDSs)

The recognition of the pharmacokinetic limitations described in the previous section has driven, in the last two decades, a substantial research effort aimed at the design of DDSs capable of solubilizing, protecting, delivering, and releasing curcumin in a controlled and, when possible, specific manner at the site of action. These systems, ranging from classic lipid vehicles to state-of-the-art nanotechnology platforms, share a common goal: to improve the bioavailability of curcumin and, in the context of NDs, to facilitate its access to the CNS. The main DDS families are systematically examined below, with an emphasis on their mechanisms of action, preclinical evidence, and, where available, clinical evidence in the field of NDs. Beyond the preclinical efficacy data, this section also highlights the manufacturing scalability, batch-to-batch reproducibility, impurity profiling, and excipient safety considerations of each DDS family, since these translational and regulatory dimensions are increasingly recognized as the principal bottlenecks to the clinical development of curcumin-based neurological therapeutics.

### 4.1. Lipid-Based Systems

Lipid-based systems represent one of the most mature and thoroughly characterized strategies for curcumin delivery. Their primary advantage lies in their ability to solubilize highly hydrophobic compounds within a biocompatible lipid matrix, thereby enhancing intestinal absorption and protecting curcumin from degradation in the gastrointestinal environment. Within this family, several subclasses with distinct characteristics can be distinguished.

#### 4.1.1. Liposomes

Liposomes are spherical vesicles composed of one or more phospholipid bilayers that enclose an aqueous core, allowing them to deliver drugs as well as proteins, nucleotides, or plasmids [27]. In the case of curcumin, which is highly lipophilic, it is incorporated into the lipid bilayer, which markedly improves its apparent solubility—from ~11 ng/mL for the free compound to approximately 1–10 mg/mL in liposomal formulations, representing a solubility increase of roughly five orders of magnitude. In preclinical pharmacokinetic studies, curcumin-loaded liposomes have been reported to increase plasma AUC by 5- to 50-fold relative to unformulated curcumin, depending on the lipid composition and particle size, and to achieve detectable brain concentrations in rodent models after intravenous administration. Preclinical studies have shown that curcumin-loaded liposomes are capable of crossing the BBB and accumulating in brain tissue, where they exert neuroprotective effects in animal models of AD. For example, in a mouse model of AD, curcumin liposomes reduced angiotensin-converting enzyme activity in specific brain regions and improved spatial memory compared to free curcumin [28]. More recently, mucoadhesive liposomal formulations for intranasal administration have demonstrated greater cerebral absorption, opening an encouraging avenue for bypassing the BBB via the nasal route [27]. Nevertheless, several limitations should be acknowledged: classical liposomes having a limited physicochemical stability, with a susceptibility to lipid oxidation, hydrolysis, and drug leakage during storage, typically requiring cold-chain logistics (2–8 °C) and limiting the shelf life to 6–12 months; rapid clearance by the reticuloendothelial system, although this is mitigated in second-generation ‘stealth’ liposomes bearing polyethylene glycol (PEG) coatings, which prolong circulation time but may themselves induce anti-PEG antibodies upon repeated administration; and batch-to-batch reproducibility remaining a significant manufacturing challenge, as small variations in the lipid composition, extrusion pressure, or hydration temperature can lead to substantial differences in the particle size distribution, encapsulation efficiency, and in vivo performance. These factors have driven the development of second-generation formulations (pegylated stealth liposomes) and active targeting ligands [27].

#### 4.1.2. Solid Lipid Nanoparticles and Nanostructured Lipid Carriers

Solid lipid nanoparticles (SLNs) are colloidal systems composed of a lipid matrix that is solid at body temperature and stabilized by a surfactant, which allows for the incorporation of lipophilic drugs. SLNs offer advantages over liposomes in terms of physicochemical stability, production scalability, and protection against degradation, although their loading capacity is limited by the tendency of the active ingredient to be expelled during the cooling and solidification of the matrix. To overcome this limitation, nanostructured lipid carriers (NLCs) were developed, which combine a solid lipid matrix with a liquid lipid component (usually a triglyceride), thereby increasing the loading capacity and reducing drug expulsion [9,29]. In a pivotal preclinical study conducted by Malvajerd et al., curcumin was encapsulated in SLNs and NLCs, and cerebral accumulation was evaluated following intravenous administration in rats (4 mg/kg). The results demonstrated that curcumin-loaded NLCs achieved a cerebral AUC0-t of 505.76 ng/g·h, markedly higher than that of SLNs (116.31 ng/g·h) and, above all, that of free curcumin (0.00 ng/g·h), underscoring the potential of these systems to deliver curcumin to the CNS [29]. Subsequent studies have confirmed that curcumin NLCs improve brain bioavailability, attenuate β-amyloid aggregation in AD models, and exert antioxidant and anti-inflammatory effects in cellular models of oxidative stress [30]. A concise comparison of the two systems—highlighting the particle size range, encapsulation efficiency, stability, and key translational challenges—is provided in Table 2.

#### 4.1.3. Nanoemulsions and Self-Emulsifying Systems

Nanoemulsions are colloidal dispersions of oil in water, stabilized by surfactants, with a droplet size typically ranging from 20 to 200 nm. Self-emulsifying systems (SNEDDs) are a preconcentrated variant that, upon contact with gastrointestinal fluids under gentle agitation, spontaneously form a nanoemulsion. Both formats exhibit an excellent ability to solubilize curcumin and have demonstrated substantial improvements in oral bioavailability [9,11,31]. Curcumin nanoemulsions have also shown neuroprotective activity in animal models of cerebral ischemia and AD, with significant reductions in markers of oxidative stress and microglial inflammation [11,12,31]. Their appeal from a translational perspective lies in the scalability of the manufacturing process (high homogenization or microfluidization pressures) and in the possibility of incorporating targeting ligands into the interfacial phase.

### 4.2. Polymer-Based Systems

Polymeric systems constitute another broad and versatile class of DDSs, characterized by a high chemical diversity, high loading capacity, and tuneable drug release governed by the selection of the polymer and its architecture.

#### 4.2.1. PLGA Nanoparticles

Poly (lactic-co-glycolic acid) (PLGA) is one of the most widely used biodegradable polymers in nanoparticle formulation, supported by a safety profile endorsed by its approval by major regulatory agencies (FDA and EMA) for use in sustained-release parenteral products. Curcumin-loaded PLGA nanoparticles are typically obtained via solvent emulsion-evaporation or nanoprecipitation techniques, exhibiting particle sizes ranging from 76 to 264 nm, encapsulation efficiencies exceeding 78%, and sustained drug release over days to weeks [11,32,33]. In the context of NDs, curcumin-loaded PLGA nanoparticles have demonstrated several beneficial effects in preclinical models: the inhibition of β-amyloid aggregation and tau phosphorylation in cellular and animal models of AD; the attenuation of oxidative stress and mitochondrial dysfunction in PD models; and an improvement in cognitive function and memory in APP/PS1 transgenic rodents [11,13,32,34]. Recent studies have explored the surface functionalization of these nanoparticles with targeting ligands or peptides (transferrin, TET-1) to increase their brain accumulation, yielding promising results in terms of specificity and the magnitude of the neuroprotective effect [11,12,32,34]. It should be explicitly noted, however, that essentially all available efficacy evidence for curcumin-loaded PLGA nanoparticles in neurodegenerative disease comes from studies in transgenic mouse models (predominantly APP/PS1, 5xFAD, and MPTP-lesioned rodents), with no published data to date from larger animal species (e.g., non-human primates) and no completed clinical trials in patients with AD, PD, MS, or ALS. This translational gap—robust rodent efficacy but no human validation—remains one of the principal obstacles to the clinical development of PLGA-based curcumin DDSs for CNS indications.

#### 4.2.2. Other Biodegradable Polymers and Polymeric Micelles

In addition to PLGA, other biodegradable polymers have been evaluated for curcumin delivery, most notably chitosan (a naturally derived cationic polysaccharide with mucoadhesive properties and the ability to transiently open epithelial tight junctions), hyaluronic acid, cyclodextrin, and alginate. Chitosan has generated particular interest owing to its capacity to enhance the paracellular transport of curcumin across the nasal mucosa—facilitating entry into the CNS via the olfactory pathway—as well as its additive effect on the inhibition of amyloid aggregation [11,34]. Polymeric micelles, formed by the self-assembly of amphiphilic copolymers in aqueous solution, feature a hydrophobic core that solubilizes curcumin and a hydrophilic shell that confers steric stability and prolongs the blood circulation time. Micelles composed of Pluronic^®^, PEG-PLA, PEG-PCL, and mPEG-PLGA are the most extensively studied in the context of curcumin delivery, with documented increases in oral bioavailability, half-life, and brain distribution of curcumin compared to the native compound in animal models [11,34].

### 4.3. Phospholipid Complexes and Phytosomes

Phospholipid complexes, also known as phytosomes when involving botanical extracts, are stoichiometric associations between an active pharmaceutical ingredient and a phospholipid (typically soybean or sunflower phosphatidylcholine) that form a supramolecular entity with an enhanced bioavailability relative to the free active compound [33]. In the case of curcumin, complexation with phosphatidylcholine modulates its interaction with biological membranes and enhances intestinal absorption, presumably via a phospholipid-mediated facilitated diffusion mechanism. The commercial product Meriva^®^ (Indena S.p.A, Milan, Italy), a curcumin phytosome formulated with soybean phosphatidylcholine at an approximate 1:2 ratio, represents one of the most extensively studied curcumin DDS in clinical settings [29]. Pharmacokinetic studies have demonstrated that Meriva^®^ increases the oral absorption of curcuminoids by a factor of >20 relative to unformulated curcumin [29]. In the context of NDs, a randomized clinical trial evaluating Meriva^®^ in osteoarthritis patients reported improvements in systemic inflammatory biomarkers, suggesting potentially relevant effects for modulating ND-associated chronic inflammation, although specific clinical trials in AD, PD, or ALS have not been conducted to date [35].

### 4.4. Solid Dispersions and Nanocrystals

Solid dispersions are systems in which the active pharmaceutical ingredient is dispersed at the molecular level within a polymeric or hydrophilic matrix, thereby markedly increasing its dissolution rate and, consequently, its bioavailability. Nanocrystals—composed exclusively of the active drug without requiring excipients—represent a particularly attractive variant owing to their high drug loading and formulation versatility [36]. Theracurmin^®^ (Theravalues Corporation, Tokyo, Japan) is a submicron colloidal dispersion of curcumin within a hydrophilic matrix, consisting of 10 *w*/*w*% of curcumin, 2% of other curcuminoids such as demethoxycurcumin and bisdemethoxycurcumin, 46% of glycerin, 4% of gum ghatti, and 38% of water. Pharmacokinetic studies in humans have demonstrated that Theracurmin^®^ increases the curcumin AUC by approximately 27-fold relative to unformulated curcumin powder, exhibiting a favourable safety profile and adequate tolerability at doses up to 400 mg/day [37,38]. In the neurological domain, a recent clinical trial evaluating Theracurmin^®^ in patients with mild-to-moderate AD reported beneficial effects on cognitive function, although the small sample size and limited study duration preclude drawing definitive conclusions. Longvida^®^ (Verdure Sciences, Noblesville, IN, USA) represents another notable example of an optimized curcumin formulation, with documented improvements in bioavailability compared with native curcumin, alongside encouraging results from a randomized clinical trial assessing cognition in healthy older adults [38,39].

### 4.5. Active Targeting Systems and Surface Functionalization

The concept of active targeting relies on modifying the surface of nanoparticles with ligands recognized by receptors or transporters preferentially expressed on target cells, thereby enhancing the drug accumulation at the site of action and minimizing the distribution to non-target tissues. In the context of ND and CNS access, transferrin receptors (TfR) and lactoferrin receptors (LfR)—abundantly expressed on BBB endothelial cells—are the most widely exploited targets [4,13]. The functionalization of PLGA nanoparticles or liposomes with transferrin or anti-TfR monoclonal antibodies has demonstrated significant increases in the brain uptake of curcumin in animal models, achieving 5- to 10-fold enhancements relative to non-functionalized nanoparticles [11,40]. Similarly, cell-penetrating peptides (CPPs, such as TAT and penetratin), specific brain endothelium-targeting peptides (TET-1), and folate or LDL receptor ligands have been employed to enhance the neuronal uptake of the encapsulated curcumin [11,12,41]. A particularly compelling aspect is the development of stimuli-responsive delivery systems that release curcumin in response to intrinsic cues from the pathological microenvironment (an acidic pH, elevated reactive oxygen species levels, and the presence of matrix metalloproteinases), enabling selective drug release within damaged tissue while mitigating systemic toxicity [10,11,34].

### 4.6. Emerging Technologies: Exosomes, 3D-Printing Systems, and Intranasal Administration

Among the most recent technologies with a high potential for CNS curcumin delivery are exosomes—extracellular vesicles of cellular origin, 30–150 nm in diameter, possessing intrinsic biocompatibility and an innate ability to cross biological barriers, including the BBB [42]. Exosomes can be loaded with curcumin using various strategies (drug incubation, sonication, and parental cell transfection) and surface-modified to enhance their target specificity. Recent studies have demonstrated that curcumin-loaded exosomes decorated with specific ligands, such as anti-DAT scFv antibody fragments, selectively accumulate in dopaminergic brain regions and exert neuroprotective effects in animal models of Parkinson’s disease [42,43]. Complementarily, stimuli-responsive nanogels (pH, temperature, and ROS) enable the controlled curcumin release within the neuroinflammatory microenvironment; copper- or zinc-based metal–organic frameworks (MOFs) offer hybrid platforms capable of co-delivering curcumin with other therapeutic agents alongside a sustained release [44]; microneedle patches incorporating curcumin encapsulated in mucoadhesive hydrogels have shown efficacy in topical and transdermal applications targeting the CNS [45]; and curcumin-functionalized carbon quantum dots (carbon dots) combine imaging and therapeutic capabilities (theranostics) within a single platform [46]. These technologies, along with 3D-printed scaffolds loaded with curcumin [47] and injectable long-acting hydrogels [48], represent the most active translational frontier in the field and should be prioritized in future research.

**Figure 4:**
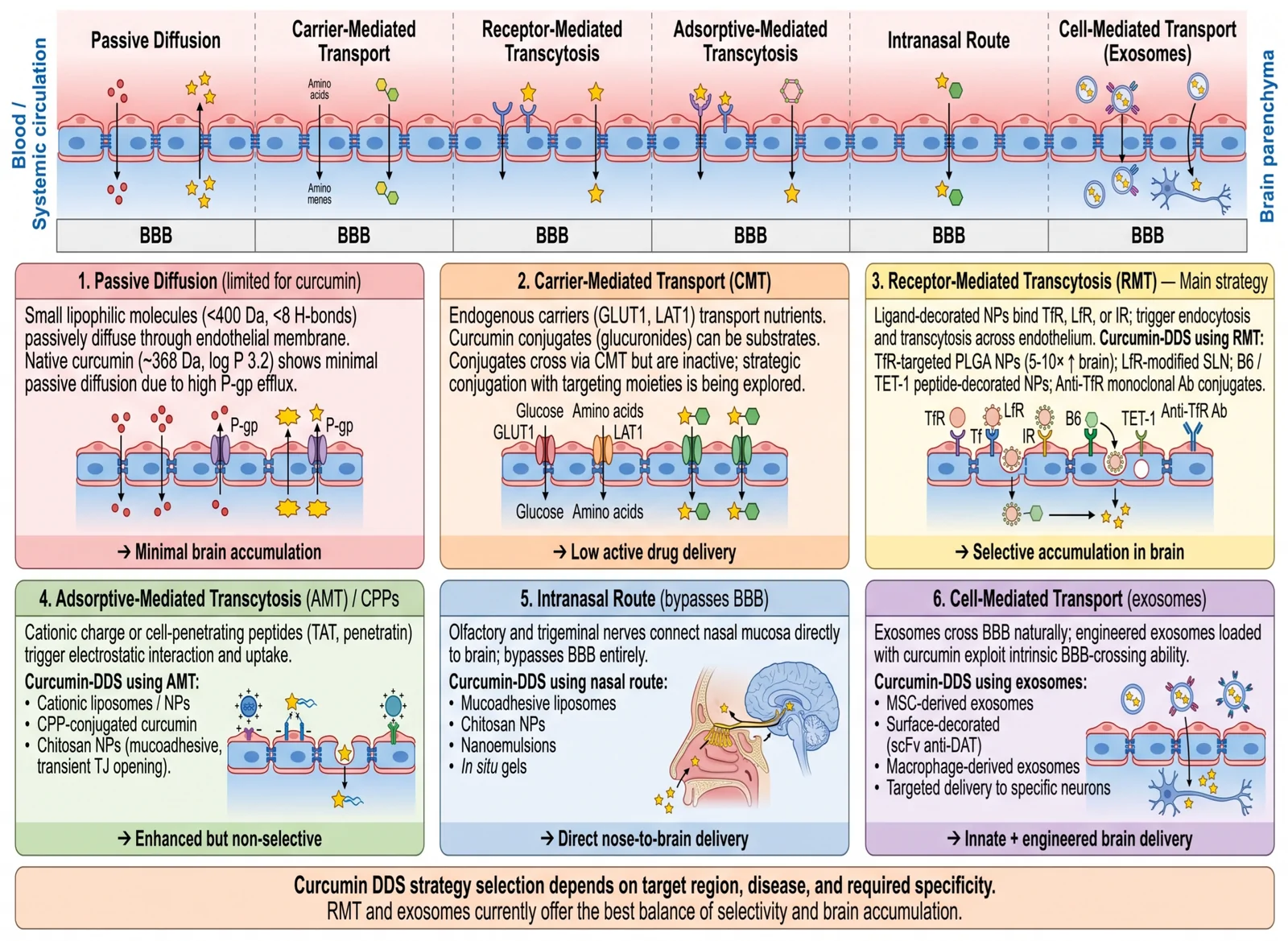
Strategies for crossing the blood–brain barrier in curcumin delivery. Because passive diffusion is limited by P-glycoprotein efflux, several active transport strategies have been developed: carrier-mediated transport (CMT) exploits endogenous transporters such as GLUT1 and LAT1 to shuttle curcumin conjugates; receptor-mediated transcytosis (RMT), currently the most widely used approach, engages transferrin, lactoferrin, or peptide ligands (TET-1, B6) on brain endothelial cells; adsorptive-mediated transcytosis (AMT) employs cationic or cell-penetrating peptides (TAT, penetratin, and chitosan); the intranasal route bypasses the BBB entirely via the olfactory and trigeminal nerves; and exosome-mediated transport leverages the innate ability of these extracellular vesicles to cross the BBB. The optimal strategy depends on the target brain region, disease, and required specificity, with RMT and exosomes currently representing the most promising approaches. Abbreviations: AMT, adsorptive-mediated transcytosis; BBB, blood–brain barrier; CMT, carrier-mediated transport; CPP, cell-penetrating peptide; CUR, curcumin; DAT, dopamine transporter; GLUT1, glucose transporter 1; IR, insulin receptor; LAT1, L-type amino acid transporter 1; LfR, lactoferrin receptor; MSC, mesenchymal stem cell; NPs, nanoparticles; PD, Parkinson’s disease; PLGA, poly(lactic-co-glycolic acid); RMT, receptor-mediated transcytosis; scFv, single-chain variable fragment; SLN, solid lipid nanoparticles; TfR, transferrin receptor; TJs, tight junctions.

To provide a comprehensive overview of the strategies currently available for curcumin delivery across the blood–brain barrier (BBB), Figure 4 summarizes the six main transport routes: passive diffusion, carrier-mediated transport (CMT), receptor-mediated transcytosis (RMT), adsorptive-mediated transcytosis (AMT), the intranasal route, and exosome-mediated transport. Among these, the intranasal route, exosome-mediated transport, and 3D-printing systems represent the most clinically promising emerging approaches, which are addressed in detail in the following subsections.

### 4.7. Synergistic Combination Strategies and Multi-Target Approaches: The Logic of Polytherapy with Curcumin

#### 4.7.1. Mechanistic Justification of Polytherapy in Neurodegenerative Diseases

Neurodegenerative diseases share a multifactorial pathogenic architecture in which at least six processes converge, with varying relative importance depending on the disease and its stage: oxidative stress, chronic neuroinflammation, mitochondrial dysfunction, pathogenic protein aggregation, the disruption of neuronal survival pathways, and the dysbiosis of the gut–brain axis (see Section 2). This convergence is responsible for the fact that the monotherapy approach, even with pleiotropic molecules such as curcumin, only partially covers the range of pathogenic targets. Curcumin robustly modulates mainly three principal axes—the Nrf2/ARE pathway, the NF-κB pathway, and the aggregation of misfolded proteins (β-amyloid, α-synuclein, and TDP-43)—but it does not directly cover equally relevant processes such as the dysregulation of neuroprotective hormonal pathways, SIRT-1-mediated synaptic dysfunction, or glutamatergic excitotoxicity. This mechanistic gap constitutes the rationale for exploring synergistic combinations in which curcumin is associated with other agents that address the gaps not reached by its pharmacological profile.

A second justification, of a pharmacokinetic nature, arises from the limitations described in Section 3. Shoba et al. [23] already demonstrated in 1998 that the coadministration of curcumin with 20 mg of piperine increases its bioavailability by 2000% in healthy volunteers, through the inhibition of UDP-glucuronosyltransferase and hepatic and intestinal sulfation.

From a technological standpoint, the fourth-generation platforms described in Section 4 (liposomes, NLCs, polymeric nanoparticles, nanogels, and exosomes) are particularly suitable for the co-encapsulation of multiple active compounds with different solubility profiles within a single particle with shared release kinetics. This approach, which Huang M, et al. have technically demonstrated to be feasible for curcumin and resveratrol in liposomes, transforms the liposomal DDS from a mere carrier of a single active compound into a targeted combination platform, simplifying the dosing regimen and reducing the interindividual variability.

#### 4.7.2. Combinations with Preclinical Evidence

Among the combinations that have been proposed, the following stand out for their potential in terms of bioavailability and efficacy:

Curcumin + piperine. The pharmacokinetic synergism described by Shoba et al. (1998) [23] represents the best-characterized combination in terms of bioavailability. Beyond the effect on absorption, both compounds share anti-inflammatory mechanisms (the inhibition of NF-κB, and COX-2) and antioxidant mechanisms (Nrf2 activation, and ROS scavenging), suggesting a potential pharmacodynamic synergy that has not been explored in depth in neurodegenerative disease models. The required piperine dose (5–20 mg) is much lower than that of curcumin (0.5–8 g), which facilitates the formulation in a single dosage unit [17].

Curcumin + resveratrol: Both polyphenols act on complementary targets: curcumin on the NF-κB/Nrf2 pathway and protein aggregation; and resveratrol on SIRT-1, the AMPK/mTOR pathway, and microglial modulation [49]. The co-encapsulation in liposomes, demonstrated by Huang et al. (2019) [50]., achieves sustained-release profiles and improved stability compared with free compounds, with documented synergistic effects in cancer models and, more recently, in neurodegeneration models. In the context of ALS, both polyphenols have shown neuroprotective effects in TDP-43 cellular models (curcumin) and in lateral sclerosis models (resveratrol), although the specific combination has been little explored until recently [50].

Curcumin + EGCG (epigallocatechin gallate): Zhou DH et al. [51] demonstrated that the combination of low concentrations of EGCG and curcumin synergistically suppresses the growth of lung cancer cell lines through cell cycle arrest, an effect that has been replicated in other tumour models. In the neurodegenerative field, both compounds share antioxidant and anti-inflammatory mechanisms, but with distinct affinity profiles: EGCG shows a greater affinity for modulating amyloid aggregation and inhibiting NADPH oxidase, whereas curcumin acts preferentially on the Nrf2 pathway. This complementarity supports their interest as a combination, although the preclinical evidence in ND models is still limited.

Curcumin + dutasteride: Proaño et al. [52] proposed dutasteride (which is a dual inhibitor of 5α-reductase isoforms 1 and 2, approved for the treatment of benign prostatic hyperplasia and androgenetic alopecia) as a candidate for the treatment of ALS, based on the following: (1) neuroprotective, antioxidant, and anti-inflammatory effects demonstrated in preclinical models; (2) documented efficacy against glutamate toxicity; (3) the restoration of altered dopaminergic activity; and (4) direct effects on TDP-43 folding, mediated both by hormonal mechanisms (the modulation of testosterone, progesterone, and 17β-estradiol) and by non-hormonal mechanisms. The combination with curcumin complements the target profile, as dutasteride provides the hormonal and glutamatergic modulation dimension absent from curcumin’s pharmacological profile. A concise comparative summary of the principal DDS platforms examined in this review, including typical physicochemical parameters, pharmacokinetic improvements, translational challenges, and representative references, is provided in Table 3.

**Table 3:** Comparative summary of the principal drug delivery systems (DDSs) for curcumin discussed in this review, including physicochemical parameters, pharmacokinetic improvements, translational/regulatory challenges, and representative references. ↑ = increase; N/A = not applicable.

Platform	Typical Particle Size	Encapsulation Efficiency	Reported ↑ in Plasma Exposure	Translational Challenges	Refs
Liposomes (classical)	100–400 nm	70–95%	5–50×	Limited stability; cold-chain storage; batch variability	[27,28]
Stealth liposomes (PEG)	100–200 nm	75–90%	10–100×	Anti-PEG antibodies; complex formulation	[27]
SLN	100–400 nm	50–90%	3–20×	Drug expulsion on storage; limited loading	[9,29]
NLC	100–300 nm	70–95%	5–30×	Complex mixtures; regulatory burden for novel excipients	[9,29,30]
PLGA nanoparticles	76–264 nm	>78%	4–25× (preclinical only)	Burst release; acidic byproducts; no neurological approval	[11,32,34]
Chitosan nanoparticles	100–300 nm	60–85%	3–15×	Polydispersity; mucoadhesive variability; limited long-term safety	[11]
Phytosomes (Meriva^®^)	100–200 nm; supramolecular	N/A (complex)	~20–30×	Supplement status, not a drug; regulatory varies by country	[33,35]
Theracurmin^®^ (solid dispersion)	0.1–10 µm; 100–300 nm	N/A (dispersion)	~30–50×	Variable bioavailability; technology-dependent	—
Active targeting (TfR, TET-1)	100–200 nm	70–90%	Variable; up to 10× vs. untargeted	Multi-step manufacturing; regulatory complexity	[11,14]
Exosomes	30–150 nm	60–85%	5–20× (rodents); limited human data	Scalable production; standardization; regulatory uncertainty	—

A quantitative summary of how the principal DDS platforms described in this section address the specific physicochemical limitations identified in Section 3.1 (low aqueous solubility of ~11 ng/mL and rapid degradation at physiological pH, t½ ≈ 10 min) is provided in Table 4. The table lists the principal mechanism of solubilization, the typical particle size and encapsulation efficiency, the reported aqueous solubility enhancement (or, where available, the plasma AUC increase relative to unformulated curcumin), and the principal translational challenges of each platform.

## 5. Applications by Disease: Status of Preclinical and Clinical Evidence

### 5.1. Alzheimer’s Disease (AD)

AD is, by far, the field of neurodegeneration in which curcumin has accumulated the most preclinical evidence, due both to the long history of research and the availability of well-characterized animal models. The molecular mechanisms through which curcumin has demonstrated effects on AD are convergent and can be grouped into four main categories. Firstly, curcumin directly inhibits the aggregation of the β-amyloid peptide, blocks the formation and elongation of Aβ fibrils, and promotes the destabilization of preformed fibrils. Ono et al. demonstrated that curcumin exhibits potent anti-amyloidogenic activity against Aβ fibril formation in vitro [30]; Lim et al. observed in the APPsw model that curcumin reduced both the amyloid plaque burden and cerebral oxidative damage [53], a finding confirmed and quantified by Yang et al., who demonstrated that curcumin binds directly to Aβ amyloid species and senile plaques, inhibits the formation of Aβ oligomers and fibrils, and reduces cerebral amyloid burden in vivo [54]; these observations were expanded upon by Garcia-Alloza et al., who demonstrated through in vivo fluorescence imaging that curcumin not only labels pre-existing amyloid plaques but is also capable of disrupting their structure and partially restoring distorted neurites in an animal model of AD [55], and by Ahmed et al., who observed that curcuminoids improve spatial memory in a rat model with β-amyloid infusion [56]. Moreover, curcumin modulates tau-associated pathology by reducing soluble tau species and the hyperphosphorylation of this protein, promotes mechanisms involved in its clearance, and attenuates neuronal and synaptic dysfunction in experimental models [57,58,59]. In addition, it inhibits the microglia-mediated neuroinflammatory response via the NF-κB/NLRP3 pathway, reducing glial activation and the sustained production of proinflammatory cytokines [60,61]. Curcumin also chelates redox-active metals (Cu^2+^, Zn^2+^, and Fe^2+^) involved in amyloid aggregation and oxidative damage [62]. Finally, curcumin activates the PI3K/Akt signalling pathway and enhances Nrf2-mediated antioxidant responses, increasing the expression of cytoprotective enzymes such as HO-1 and reducing neuronal oxidative stress [63].

From a clinical perspective, the translation of curcumin research into clinical practice for AD has been more protracted and complex than for other neurodegenerative diseases. The first randomized, double-blind, placebo-controlled clinical trial, conducted by Baum et al. (2008) using native curcumin (1–4 g/day for 6 months), showed no significant differences compared to the placebo in the primary cognitive outcomes, although the tolerability was good [64]. The observed lack of clinical efficacy has been attributed, at least in part, to the low bioavailability of native curcumin, which spurred the development of new formulations with greater systemic absorption. Among these, the Longvida^®^ solid lipid formulation has shown promising results in studies conducted in healthy older adults. In an initial randomized clinical trial, Cox et al. observed that the administration of Longvida^®^ significantly improved various cognitive domains, including working memory and sustained attention, in addition to reducing fatigue and improving mood both following acute administration and after four weeks of treatment [39]. These findings were subsequently corroborated by a second clinical trial conducted by the same group, in which 12 weeks of supplementation produced further improvements in working memory, learning, and fatigue, accompanied by a reduction in circulating triglyceride levels, although there were no significant changes in most of the other systemic biomarkers assessed [65]. However, these results come from individuals without dementia, so their extrapolation to patients with Alzheimer’s disease should be interpreted with caution; Small et al. (2018) reported beneficial effects on memory and brain amyloid accumulation as measured by PET in non-demented adults using a bioavailable curcumin formulation [66]; and Cox et al. (2015) demonstrated improvements in cognition and mood in healthy older adults using Longvida^®^ [39]. The systematic review by Voulgaropoulou et al. (2019) on the effect of curcumin on cognition, which included 32 preclinical studies and 5 clinical trials in patients with AD or healthy ageing, concluded that, although the preclinical evidence is robust, clinical trials remain limited and show inconsistent results due to their high methodological heterogeneity, differences in the formulations used, and pharmacokinetic limitations, highlighting the need for more standardized clinical trials with formulations that offer greater bioavailability [67]. The review by Chainoglou and Hadjipavlou-Litina (2020) highlighted several curcumin analogues, derivatives, and hybrids with enhanced biological activity and potential therapeutic relevance for Alzheimer’s disease [8].

Overall, AD represents the field in which the discrepancy between consistent preclinical evidence and clinical findings remains particularly evident, largely due to the pharmacokinetic limitations of native curcumin. To overcome these barriers, emerging delivery strategies—including curcumin-conjugated magnetic nanoparticles for amyloid plaque imaging [68], multifunctional nanoliposomes with brain-targeting properties [69], polymeric nanoparticles, intranasal delivery systems, and ligand-functionalized nanocarriers designed to improve blood–brain barrier transport—are being investigated as potential approaches to bridge this translational gap [11]. Furthermore, recent studies have expanded the therapeutic arsenal with curcumin derivatives designed for the in vivo imaging of amyloid plaques, as well as CNS delivery systems thoroughly reviewed by Ege [9]; a review summarizing the protective effects of curcumin against amyloid-β toxicity, oxidative stress, and mitochondrial dysfunction in Alzheimer’s disease models [70], and an integrative perspective on curcumin as a compound for cerebral anti-aging [71], consolidating curcumin and its analogues as one of the most consistent lines of preclinical and translational research in AD. Phase II/III clinical trials are urgently needed, using DDSs specifically optimized for AD, with a duration of ≥18 months, CSF exposure biomarkers (free curcumin), and combined clinical–biological endpoints (cognition + amyloid PET + plasma tau) to establish the true clinical efficacy of curcumin in this disease.

### 5.2. Parkinson’s Disease (PD)

PD is the second most studied area of application for curcumin in neurodegeneration, with a particularly large body of preclinical evidence in well-established animal models (MPTP, 6-OHDA, and rotenone) but, as noted, still very limited clinical evidence. The molecular mechanisms through which curcumin has demonstrated effects in PD can be summarized into four main pathways. To begin with, curcumin reduces the accumulation of α-synuclein and protects against dopaminergic neurodegeneration. Sharma and Nehru demonstrated that curcumin reduces α-synuclein aggregation and dopaminergic neurodegeneration in a lipopolysaccharide-induced PD model, and that these effects correlate with a sustained reduction in the striatal inflammatory response [72]. Secondly, curcumin protects mitochondrial function and attenuates mitochondria-dependent apoptosis. Pan et al. demonstrated that curcumin prevents dopaminergic neuronal loss in the MPTP model by inhibiting JNK signalling, thereby attenuating mitochondrial dysfunction and apoptosis [73], and He et al. observed that dietary supplementation with curcumin attenuates MPTP-induced neurotoxicity, reduces the activation of microglia and astrocytes, and enhances the expression of neurotrophic and neuroprotective factors, including GDNF and TGF-β1, thereby contributing to the protection of the nigrostriatal dopaminergic system [74]. Furthermore, curcumin inhibits the neuroinflammatory response by modulating microglial activation and inhibiting the NF-κB pathway, thereby reducing the sustained production of proinflammatory cytokines and promoting neuronal preservation [75]. Finally, curcumin protects against p53-mediated dopaminergic apoptosis, as demonstrated by Jaisin et al. in SH-SY5Y cells treated with 6-OHDA, showing that curcumin reduces p53 phosphorylation, decreases the Bax/Bcl-2 ratio, and significantly attenuates neuronal apoptosis [76], and Qualls et al., who observed protective effects of curcumin against toxicity induced by rotenone and salsolinol—two experimental neurotoxic insults widely used to model dopaminergic degeneration in Parkinson’s disease research [77].

From a clinical perspective, the translation of curcumin in Parkinson’s disease is, to date, the most limited of all the neurodegenerative diseases analysed in this review. A pilot randomized, triple-blind, placebo-controlled clinical trial conducted by Ghodsi et al. (2022) [78] evaluated nanomicellar curcumin (80 mg/day) as an adjunctive therapy to standard dopaminergic treatment in patients with idiopathic PD over nine months. Although curcumin supplementation did not significantly improve overall MDS-UPDRS or PDQ-39 scores compared with placebo, a significant difference was observed in the overall trend of MDS-UPDRS part III scores between groups, although this was not confirmed at individual follow-up time points. The intervention was generally well-tolerated, with gastrointestinal symptoms being the most frequently reported adverse effects [78]. Unlike in Alzheimer’s disease, the clinical evidence for curcumin formulations in PD remains very preliminary. Recently, Cai et al. and Zhu et al. have documented in MPTP animal models that curcumin exerts a neuroprotective effect in PD associated with the modulation of the gut microbiota, reinforcing the translational research on the gut–brain axis in this disease. In particular, Cai et al. show that this effect is accompanied by a restoration of short-chain fatty acid profiles, while Zhu et al. describe a dose-dependent regulation of microbiota composition and its predicted metabolic pathways [18,79].

Jayaraj et al. demonstrated that the curcumin derivative CNB-001 protects dopaminergic neurons in the MPTP model of Parkinson’s disease, supporting the development of structurally optimized curcumin derivatives [80], and Wang et al. have shown that human-umbilical-cord-derived mesenchymal stem cells, activated with curcumin, significantly improve motor symptoms and the survival of dopaminergic neurons in animal models of PD, suggesting a combined strategy of cell therapy and nutraceuticals [80]. Phase II/III clinical trials using exosomes or nanoliposomes functionalized with DAT ligands or structural derivatives of curcumin are urgently needed. These trials should have a duration of ≥12 months and include CSF exposure biomarkers and validated motor scales (UPDRS-III, and MDS-UPDRS) as primary outcomes to establish the true clinical efficacy of curcumin in this disease. Overall, the current research in PD is increasingly focused on the development of brain-targeted drug delivery systems, including exosome-based carriers, functionalized nanoparticles, and intranasal delivery approaches, to overcome the pharmacokinetic limitations of curcumin [81,82,83], and where the combination of curcumin with other strategies (mesenchymal stem cells, structural derivatives of curcumin such as CNB-001, and gene therapy with GDNF) represents the most active frontier of research [80,84,85].

### 5.3. Multiple Sclerosis (MS)

Multiple sclerosis (MS) is an inflammatory demyelinating disease of the CNS in which curcumin has shown well-documented immunomodulatory and neuroprotective effects in animal models (primarily EAE), although clinical evidence in patients is still very preliminary. The molecular mechanisms through which curcumin has demonstrated effects in MS can be organized into four main pathways. First of all, curcumin modulates the Th17/Treg balance, reducing the pro-inflammatory Th17 response (IL-17, IL-6, and RORγt) while simultaneously inducing regulatory T cells (Treg, FoxP3, and IL-10). In a pilot clinical trial with oral nanocurcumin (80 mg/day for 6 months) in patients with relapsing-remitting MS, Dolati et al. demonstrated that the treatment significantly reduced the frequency of IL-17-producing Th17 cells and showed a favourable trend compared with the placebo [86,87]. Secondly, curcumin inhibits neuroinflammatory processes in the CNS by reducing inflammatory signalling pathways, including NF-κB activation, and by limiting microglial and astroglial activation. In the EAE model, polymerized nano-curcumin attenuated neurological symptoms, reduced inflammatory and oxidative processes, and stabilized the myelin integrity [88]. These effects have been associated with the regulation of multiple inflammatory and remyelination-related pathways in experimental models of MS [89]. Moreover, curcumin promotes processes associated with myelin preservation and repair by stabilizing the myelin structure and reducing inflammatory and oxidative processes in the EAE model [88]. Lastly, curcumin attenuates cuprizone-induced demyelination in the corpus callosum, preserving the myelin content and reducing astroglial inflammation by regulating the AXL pathway [90]. Data from Koo et al. (2019) [91] in non-human primates treated with curcumin for 14–18 months confirm this impact of the polyphenol at the central level, in addition to highlighting its safety, as they demonstrated that chronic supplementation is safe and well-tolerated at the brain level and achieves significant reductions in neuroinflammation and oxidative stress markers associated with changes in the brain microstructure (decreased microscopic diffusivity and increased grey matter density) in the hippocampus and basal forebrain, which the authors interpreted as consistent with potential neuroprotective effects [91].

Clinically, the translation of curcumin into MS treatment remains at a very preliminary stage. The pilot clinical trial by Dolati et al. (2018), which evaluated oral nanocurcumin in patients with MS [86], and the randomized, placebo-controlled CONTAIN trial by Petracca et al. (2022), which investigated BCM-95^®^ curcumin supplementation as an adjunct to subcutaneous interferon β-1a therapy in patients with relapsing-remitting MS [92], represent the main clinical approaches reported to date. The first provided preliminary evidence of immunomodulatory activity, while the second showed a transient radiological signal suggestive of a possible anti-inflammatory effect, in addition to a favourable tolerability profile. However, limited sample sizes, losses to follow-up, and the absence of consistent effects on hard clinical endpoints prevent definitive conclusions from being drawn regarding the therapeutic efficacy. The experimental findings of ELBini-Dhouib et al. (2022) support a dual mechanism for curcumin in MS: on the one hand, the modulation of the innate and adaptive immune response in the periphery, and, on the other, the promotion of tissue repair in the CNS through direct effects on oligodendrocytes, astrocytes, and microglia, which could simultaneously contribute to the control of inflammation and the recovery of demyelinated tissue [89]. A line of translational research that has been particularly active in recent years is the study of the gu–brain axis in MS, where curcumin could modulate the intestinal dysbiosis associated with EAE, as suggested by studies in animal models [93] and recent reviews of the immunoregulatory effect of curcumin in EAE [94].

Overall, MS is among the diseases for which the preclinical evidence regarding curcumin is particularly consistent, but where the transition to clinical application remains the most uncertain, largely due to the same pharmacokinetic limitations of native curcumin and the lack of properly designed clinical trials. New generations of formulations—nanoparticle-polymerized curcumin [88], liposome-encapsulated nanocurcumin, curcumin derivatives such as β-D-glucuronide [93], and intranasal delivery systems designed to bypass the blood–brain barrier [81,83]—are beginning to overcome some of the pharmacokinetic limitations that have hindered the clinical translation of curcumin. Phase II/III clinical trials use these optimized formulations, lasting ≥12 months, in homogeneous cohorts of patients with relapsing-remitting MS, with biomarkers of systemic inflammation (IL-17, and Tregs), plasma neurofilament light chains (NfL), and MRI measurements of active lesions and cerebral atrophy, to establish the true clinical efficacy of curcumin in this disease.

### 5.4. Amyotrophic Lateral Sclerosis (ALS)

ALS is the neurodegenerative disease for which the evidence on curcumin has advanced most intensively in recent years, driven by both mechanistic preclinical studies and pilot clinical trials. The molecular mechanisms on which curcumin has shown effects in ALS models are grouped into three main axes. First, curcumin modulates the aggregation of SOD1. Bhatia et al. demonstrated that curcumin binds to the pre-fibrillar aggregates of SOD1 and modifies its amyloidogenic pathway, favouring the formation of less toxic aggregate species [95]; a finding recently corroborated with structural analogues such as bisdemethoxycurcumin, which effectively inhibits the formation of SOD1 amyloid fibrils in vitro [96]. Second, structural derivatives of curcumin may counteract TDP-43-associated neuronal dysfunction. Dong et al. showed that dimethoxycurcumin (DMC), a curcumin analogue, reverses the TDP-43-induced neuronal hyperexcitability in a motor neuron-like cellular model, highlighting its potential to target a key pathological mechanism implicated in both sporadic and familial ALS [97]. Third, curcumin activates the Nrf2 pathway with the induction of phase II antioxidant enzymes, whose mechanisms and evidence in ALS have been exhaustively reviewed by Carrera-Juliá et al. [98]. In our experience, the pilot clinical trial with liposomal curcumin in patients with ALS that we have recently conducted has shown good tolerability and encouraging signs regarding biomarkers of oxidative stress and mitochondrial function, findings that will be the subject of future publications by our group. Finally, curcumin derivatives such as dimethoxycurcumin normalize the neuronal hyperexcitability induced by mutant TDP-43, restoring alterations in the motor neuron excitability and action potential properties in a motor neuron-like cell model [97].

From a clinical point of view, the evidence with curcumin formulations in ALS is still limited but includes encouraging signs. Chico et al. conducted a double-blind clinical trial in ALS patients who received Brainoil (oral curcumin 600 mg/day for 6 months) and observed a favourable trend in functional progression (ALSFRS-R), along with improvements in markers of oxidative stress and aerobic metabolism [99]. More recently, Ahmadi et al. published the first randomized, double-blind, placebo-controlled pilot clinical trial of nanocurcumin (80 mg/day) as an adjunct therapy to riluzole, in which nanocurcumin was associated with a reduced risk of the combined outcome of mortality or need for mechanical ventilation compared to placebo, with an apparently greater benefit in patients with bulbar involvement, although the small sample size requires these findings to be confirmed in larger-scale trials [100]. An ongoing clinical trial with Theracurmin (NCT04499963) at Duke University is further evaluating the safety, tolerability, and potential effects of this bioavailable formulation on ALS progression markers in a 6-month open-label study in 100 patients who are taking one capsule (90 mg) twice daily [101].

The combination of curcumin with other molecules is also an aspect that, as described in Section 4.7.2, could increase efficacy. To illustrate the feasibility of this multi-target approach, our group’s IMCRELA project provides initial clinical evidence in ALS. Specifically, an oral combination of liposomal curcumin (200 mg/day) + resveratrol (75 mg/day) + dutasteride was evaluated in a randomized, placebo-controlled, double-blind trial in 74 patients with bulbar or spinal ALS. After 2 months of treatment, the combination significantly increased the baseline muscle electrical activity (*p* = 0.05 right; *p* = 0.004 left) and improved the fasciculation pattern in proximal muscles (*p* = 0.017), with good tolerability [49]. Although these results are preliminary, they constitute the first clinical evidence in ALS that a combined intervention targeting multiple pathogenic mechanisms can produce detectable neuromuscular effects, and they support the transferability of the synergistic combination strategy to other neurodegenerative diseases with multifactorial architecture.

Overall, the available evidence indicates that curcumin, either alone or in combination with other neuroprotective agents, represents a promising emerging strategy in ALS research. Advanced delivery approaches—particularly liposomal and nanoparticle-based formulations [11,30], as well as experimental cell-based delivery platforms using mesenchymal stromal cells loaded with curcumin [102]—may help overcome the pharmacokinetic limitations of native curcumin and enhance its therapeutic potential in neurodegenerative diseases, including ALS.Phase II/III clinical trials with biomarkers of CNS exposure, extended duration (≥12 months), and validated clinical endpoints (ALSFRS-R, survival, and respiratory function), which are urgently needed to establish the true clinical efficacy of these interventions.

## 6. Clinical Translation: Commercial Formulations, Human Evidence, and Regulatory Considerations

A concise summary of key human clinical trials evaluating curcumin formulations in neurological populations is provided in Table 5, listing the formulation, dose, primary endpoints, and main outcomes (efficacy signal or null).

The translation of curcumin-based advanced DDSs from the bench to bedside requires not only demonstrating clinical efficacy and long-term safety in neurodegenerative diseases, but also overcoming challenges related to formulation standardization, large-scale manufacturing, regulatory approval, and quality control before their widespread clinical implementation [103]. Table 6 summarizes the primary commercial curcumin formulations that have reached clinical development or are under advanced clinical investigation, along with their bioavailability profiles and available evidence. Overall, clinical evidence regarding curcumin formulations in neurodegenerative diseases remains limited, with human studies generally involving relatively small cohorts and reporting heterogeneous findings, thereby precluding definitive conclusions regarding the efficacy and safety [104,105]. Despite these limitations, recent systematic reviews indicate that clinical trials evaluating curcumin in cognitive decline and neurodegenerative contexts have increasingly incorporated standardized cognitive assessments and, in selected cases, disease-related biomarkers and neuroimaging measures, although substantial heterogeneity in formulations, dosing regimens, treatment duration, and outcome measures continues to limit the comparability and interpretation of the available evidence [67,105]. Immediate priorities to advance the clinical translation of curcumin include physicochemical standardization of formulations, adequately powered Phase II/III randomized clinical trials, and the incorporation of validated CSF and imaging biomarkers to improve patient selection and demonstrate target engagement, in line with the current standards for neurodegenerative drug development [106,107]. From a regulatory perspective, the translation of curcumin DDS faces specific challenges that go beyond clinical efficacy: (i) the reproducibility of manufacturing processes under Good Manufacturing Practice (GMP) conditions, including critical quality attributes such as particle size distribution, polydispersity index, encapsulation efficiency, and residual solvent content; (ii) comprehensive impurity profiling, with particular attention to process-related impurities (e.g., residual catalysts, polymerization initiators, and degradation products) and their potential neurotoxicity; and (iii) excipient safety, since some lipid excipients and surfactants used in advanced DDSs lack long-term safety data in chronic neurological indications. These regulatory dimensions, often underemphasized in preclinical publications, are increasingly being scrutinized by agencies such as the FDA and EMA, and represent a critical determinant of whether a promising curcumin formulation can realistically reach the neurology clinic. Several convergent factors plausibly explain the persistent gap between consistent preclinical efficacy and largely disappointing human trial outcomes in curcumin research. First, a fundamental pharmacokinetic mismatch exists: most animal studies use parenteral, intraperitoneal, or very high oral doses (50–500 mg/kg in rodents, equivalent to 4–40 g/day in humans) that achieve plasma curcumin concentrations 10–100× higher than those attainable in humans with the current oral formulations. Second, inter-individual variability in gut microbiota composition produces highly variable metabolite profiles, with some individuals being ‘fast metabolizers’ that produce predominantly inactive conjugates. Third, clinical endpoints in chronic NDs (ADAS-Cog and ALSFRS-R) progress slowly, requiring large sample sizes and a long follow-up to detect the modest effect sizes likely achievable with a single nutraceutical agent. Fourth, target engagement is rarely demonstrated in humans: few trials include cerebrospinal fluid (CSF) biomarkers, target occupancy by PET, or dose-finding pharmacokinetic–pharmacodynamic substudies, so the absence of efficacy cannot be unambiguously attributed to insufficient brain exposure. Fifth, the heterogeneity of commercial ‘curcumin’ products (mixtures of curcumin, demethoxycurcumin, bisdemethoxycurcumin, and tetrahydrocurcumin in variable ratios) confounds cross-trial comparisons. Together, these factors argue that the field needs rigorously standardized formulations with proven target engagement before large efficacy trials are justified, rather than additional small trials with heterogeneous products.

**Table 5:** Summary of key human clinical trials of curcumin formulations in neurological populations. ALSFRS-R = Amyotrophic Lateral Sclerosis Functional Rating Scale-Revised; ADAS-Cog = Alzheimer’s Disease Assessment Scale-Cognitive subscale; MMSE = Mini-Mental State Examination; MADRS = Montgomery–Åsberg Depression Rating Scale; LDL = low-density lipoprotein; MCI = mild cognitive impairment; n = number of patients.

Study (Author, Year)	Formulation	Dose/Duration	Population	Primary Endpoint(s)	Main Outcome	Trial Registration (ClinicalTrials.gov/ANZCTR)
IMCRELA pilot (Proaño et al., 2024) [52]	Liposomal curcumin + resveratrol + dutasteride	Curcumin 200 mg, 6 months	ALS (n = 14)	ALSFRS-R, inflammatory markers	Safety; reduced inflammatory markers; stabilized ALSFRS-R in some patients	NCT04654689 (Spain; IMCRELA, liposomal CUR + RES + DUT)
Baum et al., 2008 [64]	Curcumin capsules (unformulated)	1–4 g/day, 6 months	AD (mild–moderate, n = 34)	Safety, cognition (ADAS-Cog)	Safe, well tolerated; no significant cognitive improvement	NCT00164749 (Hong Kong; CUR + Ginkgo)
Small et al., 2018 [66]	Longvida^®^ (curcumin-lipid)	400 mg/day, 24 weeks	AD with depression (n = 80)	Depression (MADRS), cognition	No significant improvement; possible signal in subgroup	NCT01383161 (USA; Theracurmin^®^)
Chico et al., 2018 (ALS) [99]	Curcumin + galactomannan + ascorbic acid (CGA)	600 mg/day, 6 months	ALS (n = 60)	ALSFRS-R slope, survival	Slower ALSFRS-R decline; possible survival benefit	Not registered (Italy; CGA)
Ongoing Phase II/III (ClinicalTrials.gov) [101]	Various DDS (Theracurmin^®^, NovaSOL^®^)	Variable	AD, MCI, PD	Cognitive, biomarker endpoints	Mostly null results to date; ongoing trials with optimized formulations	Multiple (e.g., NCT03900530, NCT04783328, NCT04616804, NCT03549796)
Ringman et al., 2012 [108]	Curcumin C3 Complex	2–4 g/day, 24 weeks	AD (mild–moderate, n = 30)	ADAS-Cog, MMSE, CSF biomarkers	Safe; no clinical or biochemical benefit vs. placebo	NCT00099710 (USA; Curcumin C3 Complex)
Cox, K.H.M et al., 2020 [109]	Longvida^®^ (curcumin-lipid)	400 mg/day, 12 weeks	Healthy older adults (n = 60)	Cognition, mood, biomarkers	Improved working memory and mood; reduced LDL cholesterol	ACTRN12616000484448 (Australia; Longvida^®^)

**Table 6:** Commercial formulations and advanced DDSs of curcumin for neurological applications. The table includes 1st- to 3rd-generation formulations (marketed as supplements) and 4th-generation DDSs (preclinical/translational research). AUC: area under the curve; SLCP: solid lipid curcumin particle; SLN: solid lipid nanoparticles; NLC: nanostructured lipid nanoparticles; EAE: experimental autoimmune encephalomyelitis; ALS: amyotrophic lateral sclerosis; DDS: drug delivery systems; ND: neurodegenerative diseases; MOF: metal-organic framework; 3D: three-dimensional printing; AD: Alzheimer’s disease; PD: Parkinson’s disease.

Commercial Formulation	Composition/Type of System	Bioavailability Enhancement	Clinical Status	Reference
Lipid nanoparticle systems (NLC, SLN)	Colloidal systems with solid/liquid lipid matrix	Cerebral AUC 505 ng/g·h in rat for NLC vs. 0 for free curcumin	Preclinical pharmacokinetic study in rats	[29]
Meriva^®^ (Indena)	Curcumin-phosphatidylcholine (1:2)	Enhanced absorption compared with native curcumin; increased systemic exposure demonstrated in pharmacokinetic studies	Food supplement; multiple clinical trials in osteoarthritis, NAFLD, inflammation	[35]
Theracurmin^®^ (Theravalues)	Submicron colloidal dispersion (<200 nm) in hydrophilic matrix	~27× AUC vs. powder; Submicron colloidal dispersion (<200 nm) in a hydrophilic matrix	Dietary supplement; clinical trials in osteoarthritis, cognitive function, and cardiovascular safety	[38]
Longvida^®^ (Verdure)	Solid dispersion of curcumin in a lipid matrix (solid lipid curcumin formulation, SD-SLN)	Enhanced oral bioavailability and increased systemic exposure compared with standard curcumin formulations	Randomized clinical trials showing improvements in cognitive performance, mood, and fatigue in healthy older adults	[39]
MOF-Curcumin (Cu-MOF)	Metal-organic framework (Cu/Zn) loading curcumin into nanometre-sized pores	pH-dependent release; high drug loading; improved stability	Preclinical antitumor research; potential for brain tumours	[44]
Microneedles with curcumin	Microneedle transdermal patches (Zn-framework) loaded with curcumin	Sustained transdermal release; potential improvement of local bioavailability by avoiding gastrointestinal degradation and first-pass metabolism	Preclinical research (hair growth); potential application for EN under study	[45]
Scaffolds 3D of curcumin	3D-printed polymeric scaffolds (FDM) loaded with curcumin	Controlled and sustained local release; protection of curcumin within a polymeric matrix	Preclinical biomaterial application in haemodialysis membranes	[47]
Curcumin hydrogels	Multilayer hydrogels (alginate, and gelatin) with encapsulated curcumin	Sustained release; injectable or implantable application	Preclinical research; potential for local administration in CNS	[48]
Nanocurcumin (Sinapis Pharma, etc.)	Curcumin nanoparticles designed to enhance solubility, stability and oral absorption	Improved bioavailability and systemic exposure compared with conventional curcumin formulation	Preclinical evidence in neurodegenerative models; clinical studies in multiple sclerosis showing immunomodulatory effects	[86,87]
Polymerized curcumin (PCur)	Polymerized curcumin nanoparticles based on dendrosomes, a biodegradable nanomaterial designed to increase the solubility and stability of curcumin	Improves the solubility and bioavailability of curcumin compared with the conventional form, enabling greater therapeutic efficacy in preclinical models	Preclinical evidence. In an EAE model of multiple sclerosis, it reduced neurological symptoms, inflammation, and demyelination, and promoted remyelination	[88]
Liposomal curcumin (Lipocurc™, etc.)	Curcumin encapsulated in a liposomal delivery system designed to improve solubility, stability and systemic delivery	Improvement of bioavailability and systemic administration; potential neuroprotective effect mediated by antioxidant, antiapoptotic and epigenetic effects	Preclinical evidence in a Park7/DJ-1 knockout rat model of Parkinson’s disease showing improved motor deficits, reduced neuronal apoptosis and preservation of dopaminergic neurons through antioxidant and HDAC-related mechanisms	[110]
NovaSOL^®^ (AOV)	Micellar formulation (polymeric micelles)	AUC up to 185× vs. native curcumin; low concentrations of free curcumin and predominance of conjugates	Pharmacokinetic studies; limited evidence in neurodegenerative diseases	[111,112]
CurQfen^®^ (Akay)	Non-covalent curcumin-fenugreek galactomannan complex	Enhanced oral bioavailability of free curcuminoids (>45-fold increase compared with standard curcumin) and increased systemic exposure to unconjugated curcuminoids	Pharmacokinetic studies and preliminary clinical trials; specific evidence in neurodegenerative diseases remains limited	[113]
Curcumin nanogels	Nanoscale polymeric hydrogel systems (e.g., chitosan-, Pluronic-, or other polymer-based nanogels) encapsulating curcumin	Enhanced aqueous solubility, protection of curcumin from degradation, and sustained/controlled release; some systems show mucoadhesive properties suitable for mucosal delivery approaches	Preclinical research mainly in inflammatory disorders; potential applications in CNS delivery remain under investigation	[114]
Biomimetic nanoparticles	PLGA nanoparticles loaded with curcumin and coated with platelet membranes	Greater stability and sustained release; preferential accumulation in the brain lesion after intravenous administration	Preclinical research in an intracerebral haemorrhage model; inhibition of inflammation, suppression of astrogliosis, and promotion of neurogenesis	[115]
Curcumin-derived carbon dots (CurP-CDs)	Carbon nanodots synthesized from curcumin with antioxidant, anti-inflammatory, and fluorescent properties	Biocompatibility, neuroprotective activity, and potential imaging follow-up	Murine model of demyelination: reduction of myelin loss, microglial and astrocytic activation; neuroprotective potential in MS	[116]
Cavacurmin^®^ (CW8)	γ-cyclodextrin complex	Improvement in absorption compared to native, variable depending on the study	Food supplement; under investigation	[117]
BCM-95^®^ (Biocurcumax)	Mixture of curcuminoids + volatile oils of turmeric	Enhanced oral bioavailability compared with standard curcumin	Food supplements; studies in depression, osteoarthritis	[118]

## 7. Methodological Limitations and Translational Challenges

Despite the significant advances in understanding the molecular mechanisms and in the development of new curcumin formulations for NDs, substantial methodological limitations remain that must be acknowledged in order to correctly interpret the current evidence and guide future research. One major limitation is the pronounced methodological heterogeneity of preclinical studies. The different experimental models used—such as the APP/PS1 and 5xFAD models for Alzheimer’s disease, the MPT- and 6-OHDA-induced models for Parkinson’s disease, or the various active and passive variants of experimental autoimmune encephalomyelitis (EAE) in multiple sclerosis—reproduce specific aspects of the pathophysiology of each disease but do not fully reflect the biological complexity and clinical progression of human neurodegenerative diseases. In addition, there is considerable variability in the curcumin formulations employed, the administered doses, treatment duration, routes of administration, and outcome measures evaluated, making direct comparisons between studies difficult and limiting the ability to draw robust quantitative conclusions regarding the therapeutic efficacy of curcumin. These methodological differences also contribute to the limited reproducibility across studies and hinder the translation of preclinical findings into clinical practice [119,120,121].

A further class of limitations that deserves specific emphasis relates to manufacturing and regulatory scalability. Even when a curcumin DDS demonstrates robust preclinical efficacy, its clinical translation is frequently impeded by the following: (i) limited batch-to-batch reproducibility, particularly for complex multicomponent systems (e.g., surface-functionalized nanoparticles, and hybrid lipid–polymer carriers), where small variations in manufacturing parameters can lead to substantial differences in physicochemical properties and biological performance; (ii) inadequate impurity profiling, with an insufficient characterization of process-related impurities, degradation products, and batch-to-batch variability under GMP conditions; and (iii) excipient safety concerns, as some novel excipients (e.g., certain PEGylated lipids, cationic polymers, and specialized surfactants) have limited long-term safety data in chronic neurological use. Addressing these manufacturing and regulatory challenges is essential for the field to move beyond the current bottleneck, in which promising preclinical DDSs rarely progress to clinical trials in neurological populations.

The second critical limitation is the difficulty of translating the results obtained in preclinical models into clinical practice. Although most experimental studies describe beneficial effects of curcumin on oxidative stress, neuroinflammation, and cognitive performance, these findings have not been consistently reproduced in clinical trials. In this regard, the systematic review by Kehinde et al. (2025) [122] highlights that, despite the robustness of the preclinical evidence, the considerable heterogeneity in experimental designs, the formulations used, the doses administered, and the outcome measures evaluated complicates comparisons across studies and limits the extrapolation of the results to clinical practice. The authors conclude that more standardized protocols and methodologically rigorous studies are needed to facilitate the clinical translation of curcumin [122].

Thirdly, the lack of standardization in the composition, characterization, and pharmacokinetic evaluation of curcumin formulations constitutes a major obstacle to comparisons across studies. Over the past two decades, numerous formulations have been developed to improve the bioavailability of curcumin, including Longvida^®^, Theracurmin^®^, and NovaSOL^®^, which have demonstrated substantial increases in systemic exposure compared with conventional curcumin. However, the magnitude of these improvements varies considerably across studies due to the differences in formulation technologies, administered doses, pharmacokinetic study designs, and the analytical methods used to quantify curcuminoids, making it difficult to establish direct comparisons between products [41]. Furthermore, the critical reassessment conducted by Kroon et al. indicates that many claims regarding the superior bioavailability of certain formulations should be interpreted with caution, as they are often based on heterogeneous analytical methodologies and on the quantification of conjugated metabolites rather than free, pharmacologically active curcuminoids, which can lead to an overestimation of true systemic exposure [105]. This methodological heterogeneity is illustrated by the variability of bioavailability outcomes reported across pharmacokinetic studies of different formulations: Schiborr et al. demonstrated a significant increase in the oral bioavailability of curcumin from micronized powder and liquid micelle formulations relative to native curcumin, with the pharmacokinetic profiles differing between sexes [111]; Purpura et al. compared several innovative curcumin formulations and found substantial differences in the relative oral bioavailability depending on the delivery technology employed [117]; and Antony et al. reported an increased bioavailability with the BCM-95CG (Biocurcumax^®^) formulation relative to standard curcumin in a pilot cross-over pharmacokinetic study [118]. Similarly, Kumar et al. showed that the co-administration with fenugreek dietary fibre increased the bioavailability of free, unconjugated curcuminoids specifically, underscoring the importance of distinguishing free from conjugated curcuminoid fractions when comparing formulations [113]. Taken together, this heterogeneity in the analytical approach, target metabolite, and reference comparator limits the ability to establish a reliable bioavailability ranking across commercially available curcumin formulations.

Moreover, the long-term safety aspects require further investigation. Although clinical trials conducted in neurodegenerative diseases, with follow-up periods of up to approximately 12 months, indicate that curcumin has a generally favourable safety profile, with a low incidence of adverse effects, primarily gastrointestinal, the safety of its administration over longer periods remains insufficiently characterized [6,100,108]. In addition, there are specific concerns that should be highlighted: the possibility of pharmacokinetic and pharmacodynamic interactions with standard treatments (for example, potential interactions with anticoagulants such as warfarin and with other drugs metabolized by the CYP450 system); the effects on iron absorption in patients with anaemia; and the existence of reported cases of hepatotoxicity associated with the consumption of curcumin supplements, generally involving high-bioavailability formulations or patients with predisposing factors, without a dose-dependent relationship with native curcumin (clinical trials using doses of up to 8 g/day have not shown significant hepatotoxicity) [123,124,125].

In our opinion, genuine progress in the field requires implementing measures across four simultaneous fronts. Firstly, we have the development of curcumin formulations capable of improving brain exposure and demonstrating an adequate distribution within the central nervous system. To achieve this, it will be necessary to advance both strategies aimed at optimizing cerebral bioavailability and emerging localized delivery platforms, including 3D-printed biodegradable scaffolds for intracranial administration, which are currently in the preclinical stages [126]. Moreover, we have the conducting of Phase II/III clinical trials incorporating cerebrospinal fluid exposure biomarkers and validated clinical endpoints, rather than the surrogate outcomes commonly used. Thirdly, the integration of biomarkers of inflammation and oxidative stress measurable in peripheral blood or cerebrospinal fluid (CSF) would make it possible to identify patients with the greatest likelihood of responding and move toward personalized medicine. And, finally, the exploration of synergistic combination strategies (curcumin + piperine to improve bioavailability; curcumin + complementary polyphenols such as resveratrol or EGCG; and curcumin + dutasteride because of their synergistic effects on the Nrf2 pathway) may allow effective doses to be reduced while improving the tolerability profile. We believe that, without this type of coordinated approach, the risk of continuing to accumulate encouraging preclinical evidence without meaningful clinical impact will remain high.

In conclusion, curcumin remains a therapeutic candidate of enormous interest for NDs, but the maturity of the field demands a paradigm shift: fewer descriptive studies with generic formulations, and more rigorous pivotal trials with well-characterized formulations, consistent biomarkers, and adaptive designs that allow the acceleration of clinical translation. The next decade will likely be decisive in establishing whether curcumin can become a real therapeutic option for patients with NDs, or whether it will remain a complementary nutraceutical of marginal utility.

## 8. Conclusions and Future Perspective

Curcumin represents a therapeutic candidate of major interest for the treatment of NDs, owing to its pleiotropic pharmacological profile and favourable safety profile. However, its pharmacokinetic limitations—namely, its low aqueous solubility, extensive metabolism, and restricted BBB penetration—remain critical barriers that have hindered its effective clinical translation to date. The advanced DDS analysed in this review, ranging from lipidic, polymeric, and phytosomal systems to active targeting strategies and emerging technologies such as exosomes, offer partial solutions to these limitations. Accumulated preclinical evidence strongly suggests that these platforms are essential to unlocking the therapeutic potential of curcumin within the CNS.

Regarding future perspectives, as argued throughout this review, the clinical translation of curcumin in neurodegenerative diseases requires the integration of a three-pronged strategy: (1) fourth-generation DDSs with the demonstrated ability to cross the BBB and release the active compound in target brain regions (including liposomes, exosomes, nanogels, and 3D scaffolds); (2) exposure biomarkers in CSF and biomarkers of inflammation/oxidative stress in peripheral blood or CSF to identify responder patients; and (3) synergistic combinations that complementarily target the broad spectrum of pathogenic mechanisms. The IMCRELA model (liposomal curcumin plus resveratrol and dutasteride for the treatment of ALS) provides an initial clinical proof of concept for this three-pronged strategy. However, Phase II/III trials with larger sample sizes, a longer follow-up, and exposure biomarkers are needed to confirm the transferability of these findings to other neurodegenerative diseases.

## Figures and Tables

**Table 1:** Principal Phase I, Phase II, and microbiota-mediated metabolites of curcumin, with their relative abundance and implications for biological activity.

Phase	Metabolite	Relative Abundance	Biological Activity
Phase I (reduction)	Dihydrocurcumin	Minor	Partially retains antioxidant activity
Phase I (reduction)	Tetrahydrocurcumin	Major reduction product	Retains antioxidant/anti-inflammatory activity; documented neuroprotective effects
Phase I (reduction)	Hexahydrocurcumin	Minor	Partially active
Phase I (reduction)	Octahydrocurcumin	Minor	Active via Nrf2 pathway
Phase II (conjugation)	Curcumin glucuronide	Dominant (>90% in plasma)	Virtually inactive; rapidly eliminated
Phase II (conjugation)	Curcumin sulfate	Dominant (>90% in plasma)	Virtually inactive; rapidly eliminated
Microbiota-mediated	Dihydroferulic acid	Variable (diet-/microbiota-dependent)	Bioactive; antioxidant properties
Microbiota-mediated	Ferulic acid	Variable	Bioactive; antioxidant/anti-inflammatory

**Table 2:** Comparison of solid lipid nanoparticles (SLNs) and nanostructured lipid carriers (NLCs) for curcumin delivery, highlighting key physicochemical and translational parameters.

Property	Solid Lipid Nanoparticles (SLN)	Nanostructured Lipid Carriers (NLC)
Particle size range	50–1000 nm (typically 100–400 nm)	50–1000 nm (typically 100–300 nm)
Lipid matrix composition	Single solid lipid (e.g., tristearin, Compritol)	Solid lipid + liquid lipid (e.g., miglyol) blend
Drug loading capacity	5–25% (limited by drug expulsion on cooling/solidification)	10–40% (higher; liquid lipid prevents expulsion)
Encapsulation efficiency	50–90%	70–95%
Storage stability	Moderate (6–12 months at room temperature); polymorphic transitions and drug expulsion over time	Higher (12–24 months); more stable lipid matrix
Scalability of production	High (high-pressure homogenization, scalable)	High (same manufacturing processes as SLN)
Key translational challenges	Drug expulsion during storage; limited loading; unpredictable gelation risk	More complex lipid mixtures; higher regulatory burden for novel excipients; batch-to-batch variability in liquid lipid content

**Table 4:** Quantitative summary of how the principal DDS platforms address the specific physicochemical limitations of native curcumin identified in Section 3.1 (low aqueous solubility of ~11 ng/mL and rapid degradation at physiological pH, t½ ≈ 10 min). AUC = area under the curve; NLC = nanostructured lipid carriers; NP = nanoparticle; PEG = polyethylene glycol; PLGA = poly(lactic-co-glycolic acid); SLN = solid lipid nanoparticles; TfR = transferrin receptor. ↑ = Increase. References to original studies are provided in Section 4.

DDS Platform	Solubilization Mechanism	Aqueous Solubility/AUC Improvement	pH Stability	Translational Challenges
Native curcumin (reference)	None (crystalline powder)	~11 ng/mL (~30 nM) [3.1]	t½ ≈ 10 min at pH 7.4 [3.1]	N/A (baseline)
Liposomes (classical)	Incorporation into phospholipid bilayer	AUC ↑ 5–50×; solubility ↑ to ~1–10 mg/mL	Improved (lipid bilayer protects from hydrolysis)	Stability, cold-chain, batch variability
Stealth liposomes (PEG)	Bilayer + PEG corona	AUC ↑ 10–100×	Improved	Anti-PEG antibodies
SLN	Solid lipid matrix (triglyceride)	Solubility ↑ to ~100–500 µg/mL	Improved	Drug expulsion, gelation
NLC	Imperfect liquid-in-solid lipid matrix	Solubility ↑ to ~1–5 mg/mL; AUC ↑ 5–30×	Improved	Complex lipid mixtures
PLGA nanoparticles	Encapsulation in biodegradable polymer	AUC ↑ 4–25× (preclinical)	Improved	Burst release, acidic byproducts
Chitosan nanoparticles	Encapsulation + mucoadhesion	Solubility ↑ to ~1 mg/mL	Improved	Polydispersity, mucoadhesive variability
Phytosomes (Meriva^®^)	Phosphatidylcholine complex (1:2)	AUC ↑ ~20–30×	Improved	Supplement, not drug
Theracurmin^®^ (solid dispersion)	Submicron colloidal dispersion	AUC ↑ ~30–50×	Improved	Variable bioavailability
Active targeting (TfR, TET-1)	Targeted NP surface modification	Variable; up to 10× vs. untargeted	Improved	Multi-step manufacturing
Exosomes	Endogenous vesicles; bilayer + proteins	AUC ↑ 5–20× (rodent data only)	Improved	Scalable production, standardization

## Data Availability

No new data were created or analysed in this study. Data sharing is not applicable to this article.
